# Profound structural conservation of chemically cross-linked HIV-1 envelope glycoprotein experimental vaccine antigens

**DOI:** 10.1038/s41541-023-00696-w

**Published:** 2023-07-13

**Authors:** Gregory M. Martin, Rebecca A. Russell, Philip Mundsperger, Scarlett Harris, Lu Jovanoska, Luiza Farache Trajano, Torben Schiffner, Katalin Fabian, Monica Tolazzi, Gabriella Scarlatti, Leon McFarlane, Hannah Cheeseman, Yoann Aldon, Edith E. Schermer, Marielle Breemen, Kwinten Sliepen, Dietmar Katinger, Renate Kunert, Rogier W. Sanders, Robin Shattock, Andrew B. Ward, Quentin J. Sattentau

**Affiliations:** 1 Department of Integrative Structural and Computational Biology, The Scripps Research Institute, La Jolla, CA USA; 2 The Sir William Dunn School of Pathology, The University of Oxford, Oxford, UK; 3 Polymun Scientific Immunbiologische Forschung GmbH, Klosterneuburg, Austria; 4 Department of Biotechnology, University of Natural Resources and Life Sciences, Vienna, Austria; 5 Department of Immunology, National Food Chain Safety Office, Directorate of Veterinary Medicinal Products, Budapest, Hungary; 6 Viral Evolution and Transmission Unit, Division of Immunology, Transplantation, and Infectious Diseases, IRCCS Ospedale San Raffaele, Milan, Italy; 7 Imperial College London, Department of Medicine, Division of Infectious Diseases, Section of Virology, Norfolk Place, London, W2 1PG UK; 8 Department of Medical Microbiology, Academic Medical Centre University of Amsterdam, Amsterdam, The Netherlands; 9 Present Address: Department of Immunology and Microbial Science, The Scripps Research Institute, La Jolla, CA USA

**Keywords:** Protein vaccines, HIV infections

## Abstract

Chemical cross-linking is used to stabilize protein structures with additional benefits of pathogen and toxin inactivation for vaccine use, but its use has been restricted by the potential for local or global structural distortion. This is of particular importance when the protein in question requires a high degree of structural conservation for inducing a biological outcome such as the elicitation of antibodies to conformationally sensitive epitopes. The HIV-1 envelope glycoprotein (Env) trimer is metastable and shifts between different conformational states, complicating its use as a vaccine antigen. Here we have used the hetero-bifunctional zero-length reagent 1-Ethyl-3-(3-Dimethylaminopropyl)-Carbodiimide (EDC) to cross-link two soluble Env trimers, selected well-folded trimer species using antibody affinity, and transferred this process to good manufacturing practice (GMP) for experimental medicine use. Cross-linking enhanced trimer stability to biophysical and enzyme attack. Cryo-EM analysis revealed that cross-linking retained the overall structure with root-mean-square deviations (RMSDs) between unmodified and cross-linked Env trimers of 0.4–0.5 Å. Despite this negligible distortion of global trimer structure, we identified individual inter-subunit, intra-subunit, and intra-protomer cross-links. Antigenicity and immunogenicity of the trimers were selectively modified by cross-linking, with cross-linked ConS retaining bnAb binding more consistently than ConM. Thus, the EDC cross-linking process improves trimer stability whilst maintaining protein folding, and is readily transferred to GMP, consistent with the more general use of this approach in protein-based vaccine design.

## Introduction

HIV-1 vaccine design is primarily focused on eliciting neutralizing antibodies (nAb) by targeting the viral envelope glycoproteins (Env), the only target of nAb^1^. Over the past decade, a large number of broadly neutralizing antibodies (bnAbs) that target highly conserved surfaces on HIV-1 Env have been isolated from HIV-1-infected individuals, and bnAb infusion into non-human primates and human immune system mice can provide sterilizing immunity^1^. This provides proof of concept that if bnAbs could be elicited by active vaccination, they would be protective. However, Env is metastable and adopts different conformational states with implications for bnAb binding and elicitation^2,3^. This is particularly true for soluble forms of Env, which require specific stabilizing mutations to remain in trimeric form. Currently, the two leading approaches to preparing soluble, near-natively folded HIV-1 trimers for vaccine use are either cleaved between gp120 and gp41 with stability maintained by engineered disulfide bonds and other mutations (termed ‘SOSIP’), or the cleavage site replaced or supplemented by a flexible linker (here termed Uncleaved pre-Fusion Optimized, UFO). Recently a novel SOSIP trimer, ConM, based upon the sequence of all group M isolates, showed native-like morphology by electron microscopy (EM), bound most bnAbs but not non-neutralizing Abs (non-nAbs), and elicited antibodies in rabbits that neutralized pseudoviruses carrying the autologous Env^4^. Similarly, a combined SOSIP—UFO-type design of a group M consensus soluble trimer (ConSOSL.UFO, here termed ConS), was shown by EM to be well-folded in the ‘closed’ conformation and displayed bnAb and non-nAb binding similar to its membrane-anchored counterpart^5^. However, despite their relative stability in vitro, these trimers may still sample different conformational states, and their structural and antigenic stability after in vivo administration is unknown, but likely to be detrimentally affected by enzymatic and biophysical attack. Moreover, we recently demonstrated that HIV-1 Env-elicited antibody-binding to non-neutralizing epitopes on the base of soluble trimers promotes trimer dissociation. These drawbacks could be mitigated by chemical cross-linking of the trimer^6^.

Chemical cross-linking remains widely used for pathogen and toxin inactivation, and for stabilizing proteins for vaccine use and structural analysis. However, how this process affects protein structure at the molecular and atomic level, and how this impacts vaccine antigenicity and immunogenicity is poorly understood and remains empirical for vaccine use. Cross-linking endpoints for vaccine use are generally defined as infectivity reduction for inactivated pathogens^7^, or depletion of enzyme activity for toxins^8^, with mostly unknown effects on immunogenicity by comparison with the unmodified material. Moreover, we have only a crude understanding of how cross-linking influences vaccine structure and antigenicity. Monoclonal antibody (mAb) binding to hemagglutinin was reduced by formaldehyde (FA)-treatment for inactivated influenza vaccines^9,10^ and to tetanus toxin (TT) to produce the toxoid^11^. Similarly, FA or glutaraldehyde (GLA) treatment reduced pertussis toxin enzymatic and carbohydrate-binding activities^12,13^, and FA inactivation of poliovirus inhibited receptor binding^14^. Proteomic and biophysical analyses demonstrated that FA treatment of TT introduced relatively subtle molecular modifications without evidence for major protein conformational changes^15,16^. However, aldehyde inactivation of vaccines introduces additional atoms from the crosslinking agent, can reduce adaptive immune responses^17^, and in some cases such as the prototypic respiratory syncytial virus vaccine, can adversely bias adaptive immune responses driving enhanced disease upon infection^18,19^. Thus, the use of alternative, more modern cross-linking reagents without these potential adverse effects requires exploration, with the caveat that this process must be Good Manufacturing Practice (GMP) compliant for human use. Understanding the structural outcomes of chemically cross-linked vaccine antigens using high-resolution structural analysis and the effect of these modifications on the quantity and quality of ensuing adaptive immunity will allow future improvements of these approaches for the design of vaccines against other emerging and pandemic pathogens such as SARS-CoV-2.

To chemically stabilize ConM and ConS Env trimers whilst avoiding the known pitfalls of aldehyde cross-linking, we used the zero-length hetero-bifunctional carbodiamide cross-linker 1-Ethyl-3-[3-dimethylaminopropyl]carbodiimide hydrochloride (EDC). EDC activates carboxyl groups on acidic amino acid (aspartic acid, glutamic acid) side chains that react to form a covalent amide bond with proximal amines on basic amino acid (lysine, arginine) side chains, without the addition of any atoms to the molecule. Activated carboxyl groups revert to their native chemistry if cross-linking has not occurred, limiting the extent of chemical modification of EDC-treated proteins. To our knowledge, this cross-linking reagent has not previously been used to stabilize vaccine antigens. Here we have cross-linked ConM and ConS trimers using an EDC cross-linking protocol adapted to GMP. The principal finding is that EDC cross-linking almost completely preserves trimer structural integrity whilst selectively modifying antigenicity and immunogenicity, providing impetus for further application of cross-linking in the generation of highly stabilized immunogens for vaccine use.

## Results

### Biophysical, antigenic, and proteolytic resistance characterization of trimers

**Fig. 1:**
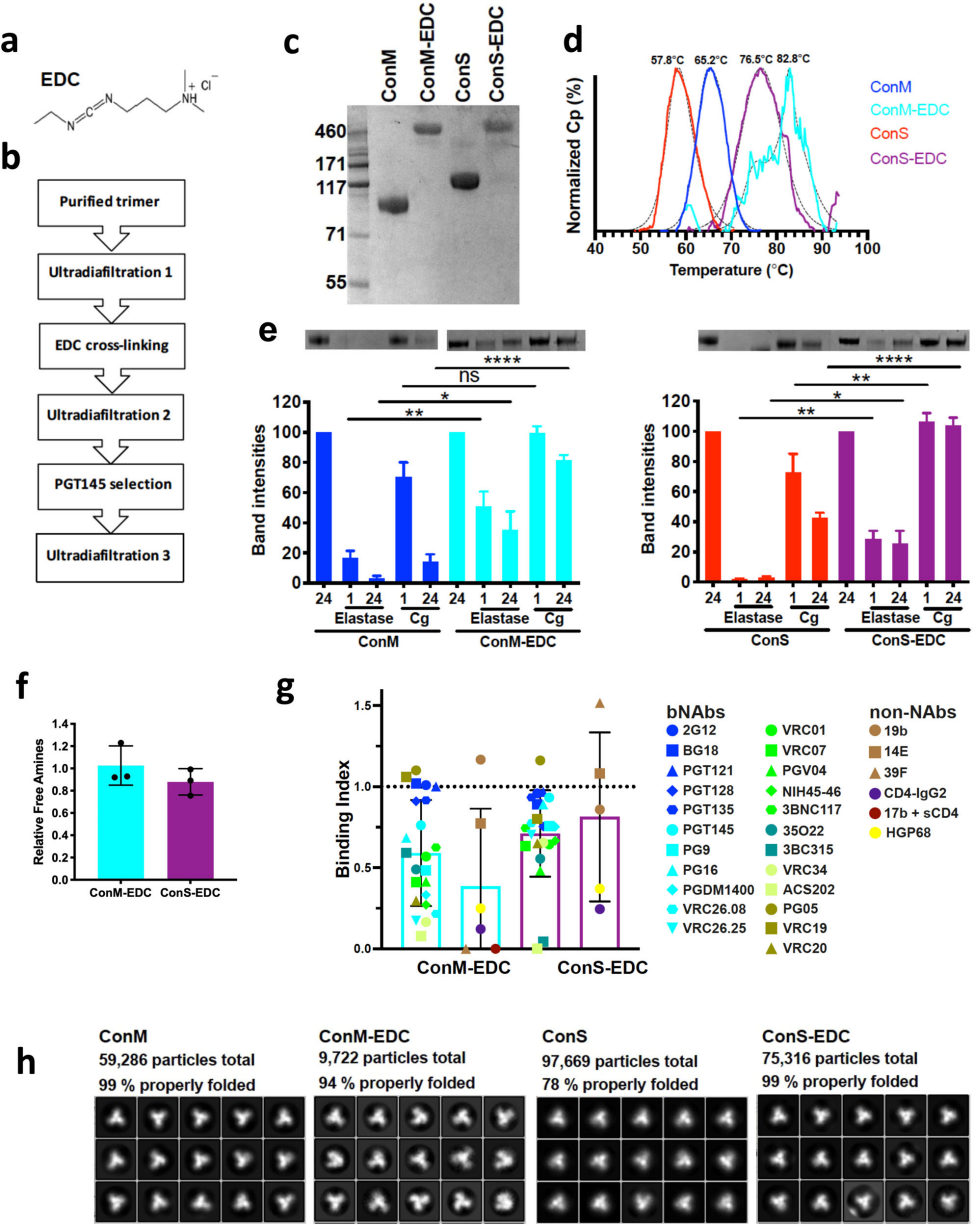
Biophysical and antigenic characterization of cross-linked GMP trimers. **a** EDC cross-linker. **b** Work-flow to GMP product. **c** SDS-PAGE reducing gel. **d** DSC analysis of trimer thermal stability. **e** Sensitivity of unmodified and EDC cross-linked trimers to neutrophil elastase and cathepsin G (Cg) attack, *n* = 3. ns = not significant; *p < 0.05; **p < 0.01; ****p < 0.0001, Mann—Whitney *U*. **f** Relative free amine content of ConM and ConS trimers compared to unmodified ConM and ConS, where 1 = no change. **g** Antigenicity of trimers using panels of bnAbs and non-nAbs, where results represent area under the curve analysis of ELISA-binding converted to the ratio of binding modified: unmodified trimer. All error bars in (**e**–**g**) represent ±1 standard deviation. **h** Negative stain EM 2D classification analyses.

ConM and ConS truncated soluble gp140 Env glycoproteins were developed and characterized as previously described^4,5^, and shown to fold into a native-like trimer configuration. ConM is based upon the sequence of all group M isolates and stabilized by an SOS disulphide bond, whereas ConS is a SOSIP—UFO combined design of a group M consensus soluble trimer (Supplementary Fig. 1). These two trimers were EDC (Fig. 1a) cross-linked using optimized conditions modified from those previously described for the BG505 trimer^20^, purified using an affinity column made with the bnAb PGT145 to select for well-folded forms, and the process translated to a GMP workflow Fig. 1b. The PGT145 quaternary epitope-specific apex-binding bnAb was chosen since it is a stringent probe of trimer conformational integrity^21^, and binds similarly to unmodified and EDC cross-linked ConM and ConS trimers (Supplementary Fig. 2). Cross-linked trimers were resistant to reducing-denaturing SDS-PAGE compared to their unmodified counterparts, which dissociated into their respective monomeric species (Fig. 1c). Because the gp120-gp41 subunits are peptide-linked in ConS, they have a higher molecular weight than ConM which dissociates into gp120 and truncated gp41 subunits. As anticipated from the SDS-PAGE outcome, differential scanning calorimetry (DSC) analysis revealed that cross-linking enhanced thermal stability by 18.7 °C and 17.6 °C for ConS and ConM respectively (Fig. 1d). Cross-linked ConM yielded an overlapping series of peaks suggesting a somewhat heterogeneous population of stabilized trimer forms.

In animal models, intramuscular administration of adjuvanted vaccine formulations rapidly recruits neutrophils (detectable within 1 h)^22^, and subsequently monocytes and macrophages^22–24^, to the injection site where they release bioactive mediators including the major leukocyte proteolytic enzymes elastase and cathepsin G^25^. We, therefore, hypothesized that adjuvanted protein vaccine immunogens will be subject to localized enzyme attack in vivo. Given the importance of correct folding of Env trimers for promoting the induction of nAb as opposed to non-nAb^26–28^, proteolytic cleavage in vivo might compromise trimer integrity and thereby limit the induction of bnAbs. To test this hypothesis, we exposed trimers in the absence of adjuvant to concentrations of elastase (200 ng) or cathepsin G (168 ng), equivalent to that estimated to be released by ~10^5^ and 5 × 10^6^ neutrophils respectively for 1 or 24 h, and analyzed their integrity by reducing SDS-PAGE. For unmodified ConM trimers this resulted in individual monomer gp120 and truncated gp41 bands, of which the gp120 band was quantified by densitometry, whereas cross-linked trimers ran intact, and these bands were similarly quantified. Unmodified ConM trimer was highly susceptible to elastase cleavage, losing >80% integrity within 1 h, and was almost completely hydrolyzed by 24 h, whereas cathepsin G had only modest effects at 1 h, but band density was reduced by ~85% at 24 h (Fig. 1e and Supplementary Fig. 3). By contrast, cross-linked ConM showed only ~50% elastase cleavage at 1 h, significantly less than its unmodified counterpart (*p* < 0.01), reducing to 65% at 24 h, again significantly less than the unmodified trimer (*p* < 0.05). ConS ran as a truncated gp140 band due to the linker peptide between the gp120 and gp41 subunits, and showed high sensitivity to elastase cleavage, with substantial band degradation by 1 h, and partial sensitivity to cathepsin G, with ~30% and ~60% reduction at 1 and 24 h respectively. As with ConM, cross-linking significantly protected the trimers from elastase degradation under every condition tested (1 h, *p* < 0.001; 24 h, *p* < 0.05), and completely prevented cathepsin attack (1 h *p* < 0.01, 24 h *p* < 0.0001). We hypothesized that trimer sensitivity to elastase and cathepsin G cleavage is principally reduced by cross-linking-enhanced protein stability, but trimer degradation could potentially also be modulated by EDC modification of amino acid side chains within the enzyme cleavage motif. However, elastase targets small hydrophobic amino acids which would not be modified by EDC cross-linking. Cathepsin G can target positively charged amino acids, but analysis of primary amine content on cross-linked compared to untreated ConM and ConS trimers (Fig. 1f) reveals undetectable change for ConM-EDC and ~10% reduction on ConS-EDC, consistent with minimal modification of lysine side chains and potential enzymatic cleavage sites within the trimer.

Cross-linking may influence trimer antigenicity with consequent effects on bnAb and non-neutralizing antibodies (non-nAb) epitopes. Trimer antigenicity was probed by ELISA analysis with panels of bnAbs and non-nAbs, and represented as the ratio of area under the curve (AUC) analysis of modified: unmodified trimer binding, such that 1 represents no change, <1 represents reduced binding to modified trimer and >1 represents increased binding to modified trimer (Fig. 1g and supplementary Fig. 2). With the exception of the V3 glycan (N332 supersite) bnAbs, which are relatively conformationally insensitive and for which binding was well maintained regardless of modification (Fig. 1g and Supplementary Fig. 2), cross-linking differentially modified bnAb binding on the two trimers. Greater disruption of conformationally-sensitive bnAb epitopes was observed for ConM than ConS, with >50% reduction in binding for bnAbs from the apex (PG9, PGDM1400, VRC26.08, VRC26.25), CD4 binding site (CD4bs: VRC07, PGV04, NIH45–46), silent face (VRC20) and fusion peptide (ACS202 and VRC34) epitope clusters. Particularly affected were the fusion peptide-specific bnAbs ACS202 and VRC34, which lost ≥80% binding. By contrast, all bnAbs with the exceptions of PGV04 (CD4bs), 3BC315 (interface), and ACS202 (fusion peptide) retained >50% binding to ConS after cross-linking. It should be noted that ConS binds fusion peptide bnAbs ACS202 and VRC34 very weakly (Supplementary Fig. 2f) since they preferentially engage cleaved trimers^29,30^. One focus of immunogen design has been to limit the accessibility of immunodominant hypervariable epitopes such as the V3 loop and CD4-induced (CD4i) surface to reduce potentially deleterious antigenic competition for B cell activation. Interestingly no reduction of 19b or 14E (V3) binding to either trimer was observed, demonstrating that these epitopes are constitutively exposed on these trimers whether native or modified. Other non-nAb epitopes (39 F, 17b, 412d, C11) required long ELISA exposure times for detection of binding, and where a signal was obtained it was largely (17b) or completely (39 F, 412d, C11) non-specific (Supplementary Fig. 2g–j).

Since ConM was more substantially impacted by cross-linking than ConS, we probed bnAb epitopes on this trimer using bilayer interferometry (Octet) to give a more quantitative binding analysis (Table 1 and Supplementary Fig. 4). The pattern of bnAb binding to modified and unmodified trimers was largely consistent with the ELISA data but revealed greater differences. Thus 35022 (interface), PGT145 (apex), and VRC43 (fusion peptide) showed reductions in *K*_D_ of 20, 17, and 38-fold respectively (Table 1). Interestingly, the dramatic loss of VRC34 binding upon EDC modification of ConM was reflected primarily in a highly reduced on-rate (>600-fold slower, Table 1), suggesting that bnAb access to its epitope was limited by trimer cross-linking rather than direct EDC alteration of the epitope, which would be anticipated to impact off-rate. This may be expected since the non-polar fusion peptide epitope for VRC34 does not contain any carboxyl or free amine groups aside from the N-terminus. sCD4-IgG2 binding to unmodified trimers was very weak and abolished by cross-linking, implying that EDC cross-linked trimers will not engage CD4 on T cells, a probable advantage for vaccine immunogenicity since CD4 T cell binding would sequester trimers, and particularly the CD4bs, from B cell recognition. Similarly, weak binding of the CD4-induced epitope-binding antibody 17b^31^ to ConM (17b did not bind ConS) in the presence of sCD4 was eliminated after cross-linking.

**Table 1:** Octet analysis of bnAb affinity for unmodified and EDC cross-linked trimmers.

bnAb	Trimer	K_D_ (M)	Kon (1/ms)	Koff (1/s)
35022	ConM	3.9 × 10^−7^	4.3 × 10^3^	8.6 × 10^−5^
	ConM-EDC	2.0 × 10^−6^	7.4 × 10^1^	8.3 × 10^−5^
PGT122	ConM	<1 × 10^−12^	7.3 × 10^4^	<1 × 10^−7^
	ConM-EDC	<1 × 10^−12^	7.8 × 10^4^	<1 × 10^−7^
PGT145	ConM	3.0 × 10^−8^	1.8 × 10^4^	3.1 × 10^−4^
	ConM-EDC	1.8 × 10^−7^	3.7 × 10^3^	5.1 × 10^−4^
VRC01	ConM	1.5 × 10^−7^	3.3 × 10^3^	4.2 × 10^−4^
	ConM-EDC	ND	ND	ND
VRC34	ConM	1.2 × 10^−8^	1.9 × 10^5^	2.2 × 10^−3^
	ConM-EDC	3.2 × 10^−6^	3.1 × 10^2^	2.9 × 10^−4^

Two-dimensional classification of trimer morphology by negative stain electron microscopy (EM) revealed that unmodified ConM and ConS trimers were 99% and 78% well-folded respectively prior to modification, and 94% and 99% well-folded subsequent to cross-linking and antibody selection (Fig. 1h). Thus, EDC cross-linking and PGT145 affinity column processing may have subtly reduced the proportion of well-folded ConM trimers, whereas the same procedure selected well-folded ConS trimers from amongst a more heterogeneous unmodified population.

In summary, EDC cross-linking and PGT145 selection of ConM and ConS trimers enhanced thermal stability and resistance to biophysical and enzymatic denaturation, whilst broadly retaining (ConM) or improving (ConS) native-like trimer morphology, but deleteriously modifying ConM, and to a lesser extent, ConS antigenicity.

### Structural analysis of trimers

**Fig. 2:**
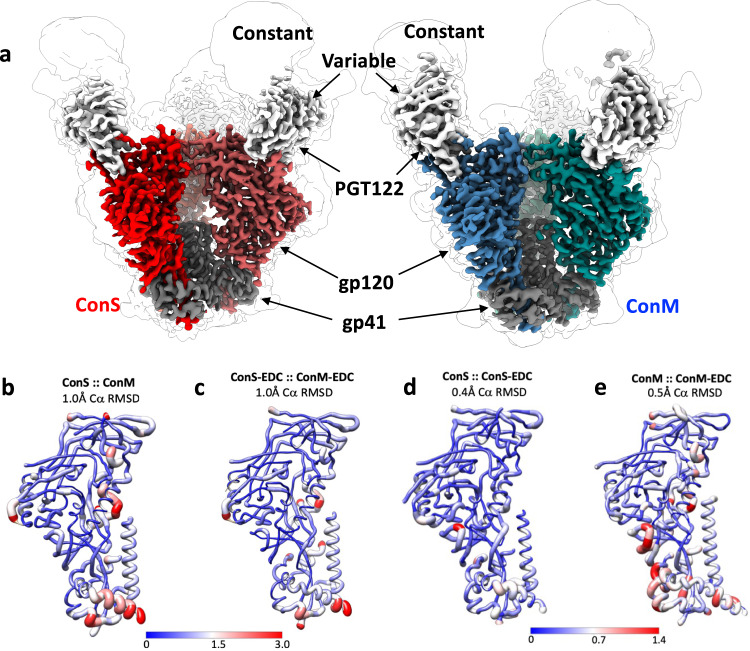
Cryo-EM analysis of ConM, ConM-EDC, ConS, and ConS-EDC. **a** Side views of unmodified ConS (red) and ConM (blue) in complex with PGT122 Fab (gray) at 3.1 Å and 3.4 Å respectively. Each cryo-EM map is shown at high (colored map) and low (light outline) thresholds to highlight the PGT122 constant domain and N-linked glycans. **b** ConM and ConS structures were superimposed, and local Cα RMSD was rendered onto the ConM structure with Chimera, according to color and to the thickness of the main chain cartoon representation. Color scales represent local Cα RMSD in Angstrom (Å). **c** Same as in **b** but comparing ConS-EDC and ConM-EDC structures rendered onto the ConS-EDC structure. **d** Same as in **b**, comparing ConS and ConS-EDC structures, rendered onto the ConS structure. **e** Same as in **b**, comparing ConM and ConM-EDC rendered onto the ConM structure.

Since preservation of HIV-1 Env trimer folding is important for maintaining conformational bnAb epitopes, in particular those dependent on quaternary conformation, it is critical that cross-linking does not adversely impact global trimer structure. Moreover, high-resolution structural information relating to cross-linking of highly conformation-dependent proteins would be helpful additional knowledge relating to the use of such stabilizing agents for understanding general principles of protein structure/function relationships. To interrogate the effect of EDC cross-linking we carried out cryo-EM analysis of trimers bound to PGT122^32^ Fab fragments, and solved the structures of unmodified and cross-linked GMP versions of ConS at 3.1 Å and 3.45 Å resolution respectively, and unmodified and cross-linked GMP ConM at 3.4 Å and 3.85 Å respectively (Fig. 2, Supplementary Table 1 and Supplementary Figs. 5–8). Negative stain EM micrographs (Supplementary Figs. 4A–7A) revealed a well-dispersed field of trimers without obvious higher-order forms or aggregates. Each Env is found in the stable closed, prefusion state, and all are highly conserved in overall structure compared to other SOSIP trimers, with an overall Cα Root Mean Square Deviation (RMSD) of 1.25 Å (ConS) and 1.2 Å (ConM) compared to BG505 (PDB 4TVP; Supplementary Fig. 9). The ConS structure is new, and the ConM cryo-EM structure is similar to the previously published^4^ X-ray structure of ConM in complex with PGT124 and 35022 (1.0 Å Cα RMSD; PDB 6IEQ), albeit at higher resolution (3.4 Å cryo-EM; 3.9 Å X-ray). Interaction of PGT122 with ConM and ConS is very similar to other SOSIP trimers, and is driven by contacts with glycans at N332 and N138 (N137 in BG505) and residues at the base of the V3 loop (Supplementary Fig. 10).

ConM and ConS trimers are both based on consensus sequences of group M isolates, and have ~91% sequence identity (Supplementary Fig. 1) and Cα RMSD between protomers of ~1 Å (Fig. 2b and Supplementary Fig. 9). Inspection of the unmodified ConM and ConS cryo-EM structures identified a number of notable features shared by these two Env designs; among these are the trimer stabilizing mutations A316W and H66R (Supplementary Fig. 11). W316 is found at the trimer apex within the V3 loop. In both structures the W316 side chain is highly ordered and mediates a cation-pi interaction with R308, and a hydrophobic stacking interaction with Y318, together stabilizing the β-hairpin structure of the V3 loop and probably impeding V3 remodeling necessary for induction of the CD4 bound state^33,34^. R66 is found within a loop in the gp120 inner domain, just beneath the apex. Interestingly, in both ConM and ConS maps, we observe density for two rotamers of the R66 side chain, with one conformation exposed to solvent and the other forming electrostatic interactions with T71 and S115. The equilibrium between these two states may underly the mechanism by which H66R inhibits CD4-induced structural changes.

### Structural Impacts of EDC Cross-linking

Since EDC is a ‘zero-length’ cross-linker, we did not anticipate the presence of additional atoms in the cross-linked structures, but the formation of individual amide bonds between proximal carboxyl and amine groups could distort local structure, and multiple cross-links might deform overall protein folding. However, comparison of the unmodified and cross-linked structures revealed a striking near identity, with a Cα RMSD of only 0.45 Å between ConS and ConS-EDC, and 0.5 Å between ConM and ConM-EDC (Fig. 2d, e). Similarly, the RMSD between cross-linked ConM and ConS trimers was also only 1 Å (Fig. 2c). Thus, within the limits of resolution, EDC cross-linking had a negligible impact on trimer structure. Overall, therefore, these data suggest that any impacts to antigenicity and bnAb binding are not a direct result of global structural or conformational changes imparted by EDC cross-linking. Moreover, this highlights a more general point that EDC cross-linking may be used to stabilize conformationally sensitive protein structure without necessarily imparting local or global distortion.

**Fig. 3:**
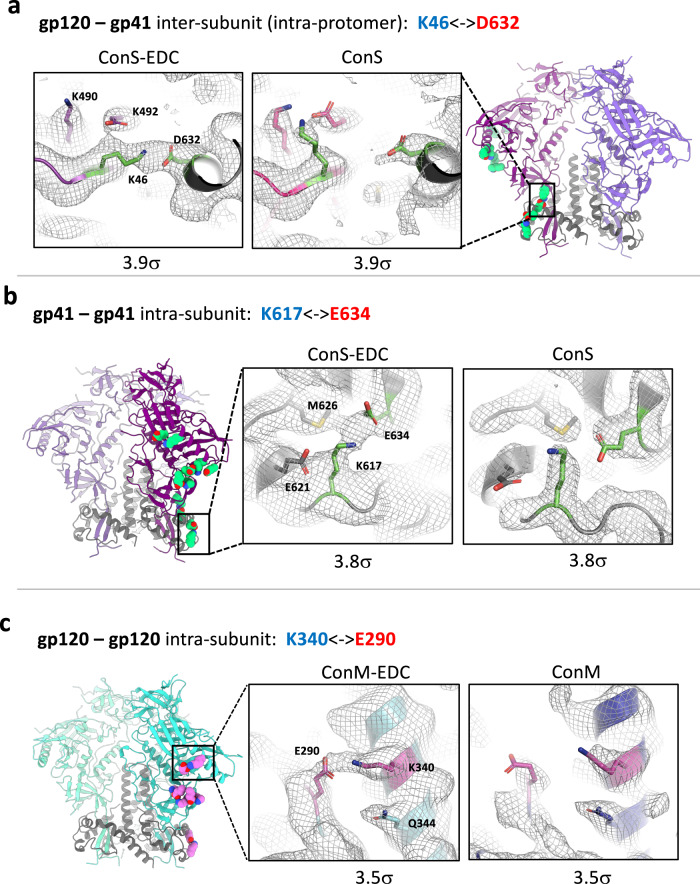
Analysis of EDC-introduced cross-links. Selected EDC cross-links observed through a comparison of modified and unmodified cryo-EM reconstructions. In **a**–**c** each map is filtered to the same resolution and the same B-factor is applied, and then visualized at the same contour level (*σ*; see methods). The overall structure is shown in ribbon representation, and the side chains of every cross-link identified are shown as green (ConS) or pink (ConM) spheres. **a** K46-D632 cross-link in ConS-EDC, which is a gp120-gp41 inter-subunit cross-link within the same protomer. The insets show a zoomed-in view of the K46-D632 cross-link, with cryo-EM density displayed as a mesh. **b** Same as in **a**, for the K617-E634 intra-subunit cross-link in ConS-EDC gp41. Note that the ribbon structure at left is rotated ~90° counterclockwise (as viewed from the top) from **a**. **c** K340-E290 intra-subunit cross-link in ConM-EDC gp120.

Throughout the ConS and ConM structures there are numerous potential EDC cross-linking sites, in which an aspartate or glutamate side chain is in proximity to, or directly interacting with, a lysine side chain (Supplementary Table 2). However, as a cryo-EM reconstruction represents an ensemble of many thousands of particles, and in this case contains C3 symmetry, only high-frequency events will be observed in the final map. Strikingly, close inspection of the ConS-EDC and ConM-EDC density maps, and careful comparison with their unmodified counterparts, revealed a series of high-confidence cross-links. Observed cross-links are listed in Supplementary Table 2, and examples are summarized visually in Fig. 3. Most were intra-subunit cross-links within gp120, with an inter-subunit gp120-gp41 (intra-protomer, Fig. 3a) and an intra-subunit within gp41 (Fig. 3b). Inter-subunit (gp120-gp41) cross-links such as K46-D632 (Fig. 3a) will stabilize quaternary structure of the complex consistent with the SDS-PAGE analysis (Fig. 1d). This should increase stability and in vivo lifetime of the ‘closed’ state of the trimer, thus maintaining bnAb epitopes but reducing exposure of non-nAb epitopes such as the V1V2 and V3 loops and the CD4i surface^20,35–37^. Since each cross-link is in essence a “molecular staple”, even intra-subunit cross-links (Fig. 3b, c) will act to stabilize secondary and tertiary structure, increasing overall molecular stability and potentially reducing exposure to protease attack.

Structural analysis informs antibody binding, allowing comparison between unmodified and cross-linked antigens. Modification of antigenicity may result from global changes in trimer folding, which we have shown is not the case. Thus, changes will most probably be local, and will result from the introduction of cross-links within or proximal to the epitope, and potentially also the change in charge at the site of cross-linking arising from amide bond formation that eliminates the previous negative charge of the reacted carboxylate group^38^. However, inspection of the epitope cluster most heavily antigenically modified in ConM, the fusion peptide, (Fig. 1f, Supplementary Figs. 1, 2 and Table 1), revealed no obvious cross-links. E87 in gp120, which is reported to be critical for ACS202 binding^29^, may conceivably be modified by EDC in ConM reducing bnAb engagement. Nevertheless, this would not explain loss of VRC34 binding to the same region of ConM, as this bnAb does not include E87 in its epitope^30^. Therefore, it seems most likely that fusion peptide exposure is prevented by locking the trimer into a ‘closed’ form. By contrast, whilst none of the CD4bs, apex, and interface epitopes analyzed contained obvious cross-links, all contained acidic residues that could form heterogenous low-frequency, unobserved cross-links with proximal lysines (Supplementary Fig. 12), potentially resulting in reduced affinity which would be consistent with the modified binding curves (Supplementary Fig. 2) and reduced *k*_D_ values (Table 1). An alternative is that the quenching step to eliminate unreacted O-acylisourea intermediates in the absence of proximal primary amines might form an amide bond with quenching agent (glycine), the added glycine residues disrupting antibody epitopes. However, there is no evidence for this since no extra density was observed on aspartate or glutamate side chains (Supplementary Fig. 12), suggesting that if it does occur, it is likely to be a rare event. Moreover, as mentioned previously, EDC cross-linking primarily influenced bnAb on-rate, strongly implying reduced epitope exposure rather than a direct impact on epitope chemistry.

Taken together, these data demonstrate a striking conservation of structure in cross-linked trimers with highly localized and epitope-specific modulation of bnAb binding evidenced by high-resolution structural analysis.

### Immunogenicity of cross-linked trimers

**Fig. 4:**
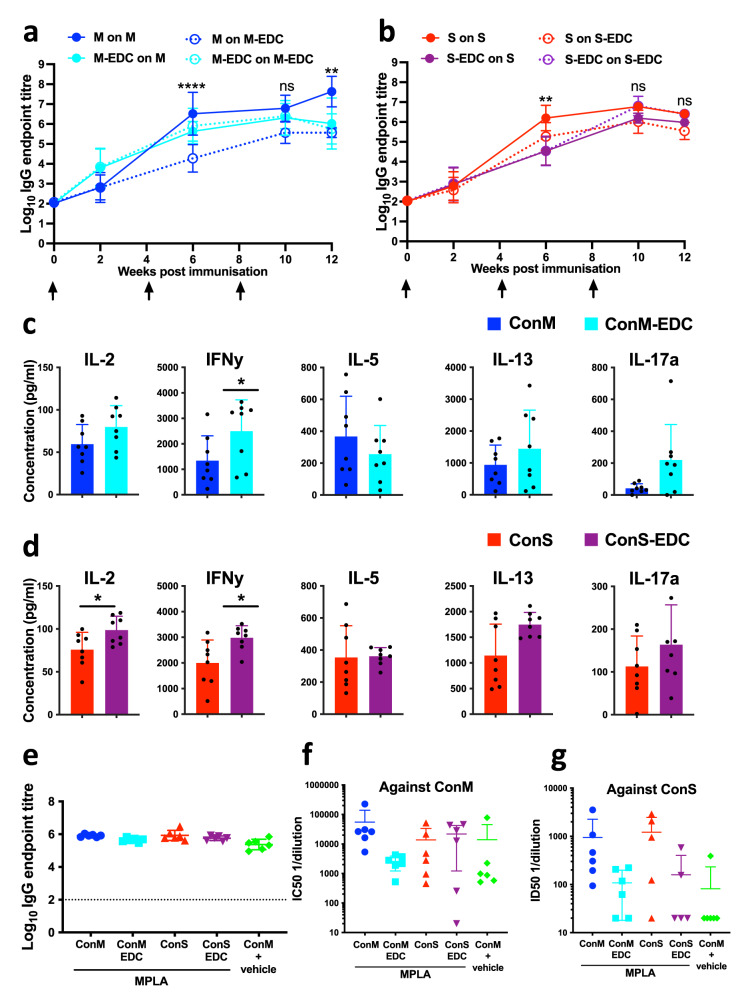
Antigenicity and immunogenicity of unmodified and cross-linked trimers. **a**, **b** Mouse serum IgG responses to immunization with (**a**) ConM (M) or ConM-EDC (M-EDC), or (**b**) ConS (S) or ConS-EDC (S-EDC), assayed against unmodified (M or S) or cross-linked (M-EDC or S-EDC) versions of the protein, where *n* = 8 mice, 4 mice/group from *n* = 2 pooled independent experiments. **p < 0.01, ****p < 0.001, one-way ANOVA with Sidak’s multiple comparison correction. **c**, **d** Mouse splenocyte antigen-specific cytokine release measured by multiplex bead array after homologous unmodified ConM (**c**) or ConS (**d**) in vitro restimulation. Data pooled from *n* = 2 experiments, datum points represent individual mice. *p < 0.05, Mann—Whitney *U*. **e** Endpoint ELISA titers of rabbit antiserum at week 12. **f**, **g** Neutralization activity against homologous and heterologous ConM (**f**) and ConS (**g**) env pseudoviruses represented by the reciprocal serum dilution giving 50% inhibition (IC_50_). All error bars represent ±1 standard deviation.

To probe antigen-specific adaptive immune responses, mice were immunized with 10 µg trimer/dose formulated in 10 µg/dose MPLA adjuvant at the times indicated by the arrows (Fig. 4a, b), and antibody kinetics analyzed by ELISA. Serum from each group was assayed for antigen-specific IgG against either unmodified or EDC cross-linked homologous trimers at the times shown. Unmodified ConM elicited an IgG response that reacted equivalently with unmodified and cross-linked ConM at week 2, but which then diverged after the prime showing significantly greater reactivity against unmodified compared to cross-linked ConM at 6 weeks (*p* < 0.0001) and 12 weeks (*p* < 0.01), Fig. 4a. Similar results were obtained for ConS (Fig. 4b), with sera from ConS-immunized mice giving significantly higher titres on ConS compared to EDC cross-linked ConS at week 6 (*p* < 0.01). These data suggest that epitopes available on unmodified trimers, potentially as a result of trimer opening and/or dissociation in vivo, are less represented on cross-linked trimers, which are unable to open and dissociate into their components. By contrast, sera from mice immunized with cross-linked ConM and ConS trimers gave very similar titres on both unmodified and cross-linked homologous trimers, consistent with epitopes available on the cross-linked trimers being equivalently presented on the unmodified trimers in the ELISA format. Differentials in antigen-specific antibody responses may be a consequence of antigen quantity or quality directly affecting the B cell response, or alternatively may reflect altered T cell help. Since cross-linking might modify antigen processing and peptide presentation to T helper (Th) cells, we evaluated antigen-specific responses to immunization. Spleens were harvested at week 14 and splenocytes restimulated in vitro with unmodified homologous antigen for 3 days, followed by analysis of cytokine release. In vitro ConM restimulated splenocytes from mice immunized with unmodified or cross-linked ConM trimer showed a non-significant trend towards enhanced IL-2, IL-13, and IL-17a but a significant (*p* < 0.05) increase in IFNγ release in cross-linked compared to unmodified trimer-immunized mice (Fig. 4c). Similarly, restimulation of splenocytes from mice immunized with cross-linked ConS trimer resulted in significantly increased IL-2 and IFNγ (*p* < 0.05) suggesting a Th1-type bias, and modest trends towards increased IL13 and IL17a release. Whilst these differences are intriguing, they do not lead to any obvious cytokine pattern that might be interpreted in terms of improved functional antibody outcomes.

Toxicity analysis of the GMP product required immunization of rabbits with trimer (100 µg) co-formulated with MPLA adjuvant (500 µg), according to the regimen in Supplementary Fig. 13a, b. IgGs purified from rabbit sera were titrated onto ELISA wells coated with the antigens shown, and titration curves generated (Supplementary Fig. 13c–f). Binding curves showed no significant differences between groups in kinetics of IgG elicitation, and endpoint titers determined at week 12 were almost identical between all adjuvanted groups (Fig. 4e). Thus, differences noted with the mouse immunizations were not recapitulated in the rabbits, potentially because the much higher (20-fold) dose of antigen used may have masked differences. Week 12 rabbit sera were assayed against infectious molecular clones (IMCs) expressing Envs from ConM and ConS in a TZM-bl assay, and results expressed as reciprocal serum dilution yielding 50% inhibition (IC_50_). Sera from rabbits immunized with unmodified ConM neutralized ConM (Fig. 4f) and ConS (Fig. 4g) IMCs approximately 30-fold and 10-fold more respectively than sera from animals that received cross-linked ConM, although the substantial variation within groups resulted in a lack of statistical significance. Similarly, cross-linked ConS trimer showed reduced autologous neutralization on ConS IMC, but again this was not significant. Since both ConM and ConS Envs generate relatively neutralization-sensitive Tier-1-type pseudoviruses^4,5^, most of the serum neutralization activity observed is likely to be against the V1V2 loops, as previously described^4^. Indeed, the inclusion of a homologous soluble trimer mutated to inactivate CD4bs engagement (D6368R) in a previously described neutralization inhibition assay^4^, resulted in complete elimination of neutralization by all sera, whereas the same trimer backbone with a heterologous V1V2 loop had no obvious inhibitory activity with any of the sera (Supplementary Fig. 14). Therefore, the reduction in serum neutralization most likely represents either beneficial masking of these structures by cross-linking V1V2 within the trimer context, or local EDC modification of the specific epitopes recognized by these Tier-1 virus nAbs. We suspect that the first hypothesis is the more likely as the animals will mount a polyclonal response to these epitopes, and so epitope-specific modification seems less likely than domain masking. Heterologous Tier-2 virus neutralization was not tested since the short immunization regimen was designed as a toxicity study and was not optimized to elicit nAb, so we did not anticipate broadly neutralizing responses.

In summary, immunization of mice and rabbits with GMP EDC cross-linked trimers formulated in MPLA adjuvant revealed broadly similar kinetics of antigen-specific IgG induction in mice and rabbits between unmodified and cross-linked trimers, with a modestly enhanced Th1-biased cytokine response in mice against the cross-linked trimers. Trimer cross-linking reduced induction of autologous virus neutralization in rabbit sera potentially via masking of V1V2 epitopes. Given the major species-specific BCR germline configurations between species, our rationale for carrying out small animal immunogenicity analyses was not designed to probe the ability of these immunogens to elicit bnAbs, but to yield a broad-brush view of adaptive immune responses linked with a safety endpoint. Experimental medicine trials will be required to determine whether these cross-linked antigens are superior immunogens compared to their unmodified counterparts.

## Discussion

Structural analysis of complex antigens has been facilitated over the past decade by the use of cryo-EM single particle analysis, allowing for near-atomic resolution. This has led to the determination of a variety of structures such as those here, which provide a detailed snapshot of the effects of chemical cross-linking on two GMP experimental vaccine immunogens destined for clinical use. The most striking outcome from the present analysis is the almost complete conservation of trimer structural integrity after robust trimer stabilization by cross-linking with EDC, which was unexpected. Surprisingly, however, the structural conservation is somewhat dissociated from antigenic conservation, with many bnAbs binding less well to the cross-linked trimers, particularly in the case of ConM. It is currently unclear why there is a substantial loss of bnAb epitopes on ConM given the high degree of structural homology, and further work is required to understand this. By contrast, ConS showed a more conserved antigenic profile after cross-linking, suggesting that the use of EDC would have to be trialed empirically for cross-linking highly conformationally sensitive glycoproteins such as HIV-1 Env.

To our knowledge, only one other study has investigated the structure of a cross-linked vaccine immunogen compared to its un-crosslinked counterpart at high resolution^37^. In this previous study, we showed that glutaraldehyde, a 5-carbon homo-bifunctional amine cross-linker, introduced cross-links into the prototypic SOSIP trimer BG505, which subtly modified global molecular conformation, and eliminated bnAb binding to quaternary epitopes at the trimer apex^37^. However, that study revealed that GLA added extra atoms to the structure with the added risk of creation of immunogenic neoepitopes, and moreover did not analyze GMP-produced material and hence is distal from translation to human use.

Our current results reveal that chemical cross-linking of two M group consensus sequence-derived Env trimers substantially enhanced biophysical stability. It is widely accepted that enhancing the stability of subunit immunogens, particularly those with metastable characteristics such as HIV-1 Env, is likely to reduce exposure to immunodominant non-neutralizing epitopes such as the V1, V2, and V3 loops^33,34,39,40^. In this respect, the inter-subunit cross-link K46-D632 may be particularly relevant since as it is likely to substantially restrict molecular movement. However, it is likely that intra-subunit cross-links and modifications will also play a role in restricting molecular movement via secondary and tertiary structure stabilization. Additional benefits of stabilization may also include increased in vivo lifetime, particularly in the presence of antibodies that trigger disassembly of non-cross-linked trimers^6^, and potential resistance to proteases found at sites of inflammation such as at vaccine injection sites. The local in vivo milieu after administration of vaccine formulations has not been well studied, but is likely to be hostile to proteins in terms of proteolytic attack, particularly when the immunogen has stimulated an inflammatory environment containing cells such as neutrophils that release highly active proteases such as elastase^22–24^. Our models of protease attack, whilst limited by the in vitro milieu and lacking the presence of adjuvant which might directly modulate protease activity, revealed that unmodified trimers were sensitive to cleavage by elastase, and to a lesser extent by cathepsin G, but that cross-linking significantly enhanced protease resistance. Measurement of antigen protease sensitivity over 1 and 24 h seems relevant, since a recent study suggests that antigen may persist at the site of intramuscular administration for several days in an adjuvant-dependent manner, and its elimination can substantially reduce ensuing adaptive immune responses^24^. Moreover, antigens are archived for weeks or longer in the form of immune complexes on follicular dendritic cells for antigen presentation to B cells in germinal centers^41^. In the case of HIV-1 Env and other highly conformational antigens, it would be most important for antigen to retain conformational integrity over time despite being complexed with antibody or complement, and cross-linking may facilitate this. Future studies will be aimed at characterizing the in vivo fate of conformationally-sensitive immunogens such as soluble Env trimers by the use of highly conformation-dependent bnAb probes. Moreover, we suggest that given the high degree of structural conservation between the unmodified and EDC cross-linked trimers, this approach could be applied to next-generation immunogens such as germline-targeting trimers^42^.

Whilst the minimal modification of trimer structure upon cross-linking suggests that immunogenicity may not be substantially affected, the complexity of the immune system is such that this is impossible to predict. We, therefore, carried out immunization of mice with the unmodified and cross-linked trimers formulated with the TLR4 agonist adjuvant MPLA. Interestingly, quantitative differences were seen in mouse T cell cytokine responses, with significantly increased titers of IFNγ (ConM-EDC and ConS-EDC) and IL-2 (ConS-EDC), compared to unmodified trimer, suggesting enhanced Th1-type responses. It is unclear why cross-linking might elicit these increases in cytokine response, particularly in light of the minimal structural changes observed, however cross-links and other EDC modifications made to acidic residues may alter antigen processing and presentation to T cells resulting in cytokine modulation. These modulated cytokine responses did not appear to result in any obvious differences in outcome for B cells, given the similar antigen-specific IgG responses. Small differences in antigen-specific IgG responses were observed in mice, but these normalized between the unmodified and cross-linked groups after the first (ConM) and second (ConS) boosts respectively. Rabbit immunizations were designed to test formulation toxicity and so were not optimized for eliciting neutralizing antibody responses. Nevertheless, autologous and heterologous Tier-1a (ConM) and Tier-1b (ConS) pseudovirus-neutralizing responses were observed. Interestingly both autologous (in ConM and ConS sera) and heterologous (in ConM sera) responses tended to be reduced by cross-linking, suggesting that Tier-1 type neutralization epitopes were being masked or otherwise compromised, and the V1V2 loops appear the most likely targets in this respect. Since one of the goals of immunogen design is to focus immune responses away from regions that may compete for the production of bnAbs, such reductions in Tier-1 neutralization may be helpful. Future studies will address the target specificity of these responses and the mechanism of their reduction in cross-linked trimers.

The ability to structurally analyze complex molecules such as the HIV-1 Env trimer by cryo-EM allows the unprecedented high-resolution analysis of post-translational modifications such as chemical cross-linking, which until recently have not been interrogated in vaccine antigens. This information will enable a more rational approach to immunogen stabilization, particularly for molecules that are structurally and/or conformationally metastable. Equally, the proof of concept shown here for the use of EDC in manufacturing a GMP immunogen, currently in experimental medicine trials, paves the way for the use of this and other newer cross-linking reagents in vaccine manufacture. Indeed, the EDC treatment added only two additional steps to the GMP process. Since EDC adds no additional atoms to the cross-links, unlike, for example, glutaraldehyde, there will be minimal risk of generation of neoepitopes with associated concerns of antigenic modification potentially associated with cross-reactivity and autoimmunity.

## Methods

### Antibodies and ligands

Antibodies VRC01^43^, PGT145^32^, 2G12^44^, PGT122^32^, 19b^45^, 35022^46^, ACS202^47^, and VRC34^30^ were expressed in freestyle 293 F cells under serum-free conditions and purified by protein A chromatography (Thermofisher) following manufacturer’s instructions. Soluble CD4 (sCD4)^48^, CD4-IgG2^49^, 15e^45^, F105^45^, 17b^31^, 19b and PG16^50^ were from the IAVI Neutralizing Antibody Consortium. Fragments antibody-binding (Fabs) VRC01, PGT122, PGT145, and 35022, used in BLI-Octet studies, were expressed in Freestyle 293 F cells under serum-free conditions and purified using a HiTrap KappaSelect column (GE Life Sciences #17545812, for Fab). The column was washed with phosphate-buffered saline and eluted with 0.1 M glycine pH 2.7. The fractions were concentrated, and the buffer was changed to 20 mM sodium acetate pH 5.5. The Fab was loaded into a Mono S column and was eluted with a 0–60% linear gradient of 1 M sodium chloride in 20 mM sodium acetate pH 5.5 buffer. The Fabs were concentrated and stored in 20 mM sodium acetate pH 5.5, PBS, or TBS at 4 °C or at −80 °C. VRC43 Fab was a kind gift from P Kwong and the VRC. Where required, antibodies were biotinylated using EZ-link NHS-LC-Biotin according to the manufacturer’s instructions (Thermofisher), or were attached to cyanogen bromide-activated agarose using the manufacturer’s protocol (GE Healthcare).

### Generation of CHO cell lines for stable expression of ConM and ConS Env trimers

Stable Chinese Hamster Ovary (CHO) cell lines expressing ConM and ConS Env trimers were generated by transfection of a parental CHO K1 host cell line using BAC (Bacterial Artificial Chromosome) vector technology^51^. The transfection strategy for ConM (but not ConS) involved co-transfection (ratio 1:1) of a second BAC vector harboring the gene coding for the human pro-protein convertase (pc) furin for efficient cleavage of the Env precursor in the trans-Golgi network (TGN)^52^. Positive transfected cell pools for both trimers were selected in serum-free medium (CD CHO (Gibco), supplemented with selection medium (SM, 8 mM L-glutamine and phenol red, with 0.5 mg/mL G-418, Sigma). Cell pools were maintained at 37 °C, 5% CO_2_, and 80% humidity in a shaking incubator (Kuhner) at 125 rpm, and regularly checked for onset of cell growth until cell viability reached >95 % with steady growth. ConM and ConS expressing cell pools were expanded to 125 mL shake-flasks (Corning) and were split every 3–4 days to starting cell concentrations of 2–3 × 10^5^ cells × mL^−1^, in SM. Cell concentrations and cell viabilities were monitored using a Bioprofile CDV cell counter (Nova Biomedical, US) and a Multisizer 4e (Beckman Coulter, US). At each passage, supernatant samples were collected for quantification of ConM and ConS Env titres by PGT145 binding ELISA.

### Isolation of ConM and ConS Env trimer clonal lines

Clonal lines of ConM and ConS transfected cell pools were obtained through limiting-dilution single-cell cloning. Cell pools were diluted to a limiting cell concentration of 1 cell per 40 µL well aliquot in a conditioned single-cell-cloning medium (1:1) mixture of SM, and 0.2 µm filtered CHO medium from a 3-day passaging harvest from cultivation of the parental CHO K1 host cell line, supplemented with rhAlbumin (Sigma Aldrich), rEGF (Repligen) and rTransferrin (Merck). Diluted cell suspensions were plated into 384-well plates (Corning), centrifuged at 200 x g for 5 min, and imaged with a CellMetric (Solentim) imaging device to capture single-cell plating events, and to follow the outgrowth of clonal cell populations. Clonal ConM and ConS cell populations were then expanded to 96-well plates (Thermo Scientific) and evaluated for cell growth using the CellMetric imaging system, and expression of ConM and ConS Env trimers was measured by PGT145 ELISA at the end-point of two consecutive passages. Based on expression levels and cell growth, 20 ConM and ConS lead clones were selected, expanded into 25 cm² tissue culture flasks (Greiner), and cultivated for three consecutive passages. Sampling at each passage end-point (every 3rd to 4th day) included measurement of cell concentration and viability. Further, cell-free supernatant was collected for analysis of Env trimer expression by PGT145 ELISA. Additionally, cell pellets of the 20 ConM lead clones were analyzed for the co-transfected hFurin construct by standard end-point PCR and hFurin specific primers. Based on cell growth and PGT145 ELISA expression data, ConM and ConS Env cell-specific productivity was calculated on the basis of which lead clone number was further reduced to eight, which were then cryo-preserved and stored in liquid nitrogen.

### Evaluation of ConM and ConS Env trimer expressing CHO lead clone candidates

In order to identify optimal clones to enter the GMP manufacturing pipeline, small-scale shake-flask fed-batch experiments were performed. Fed-batches were performed in ActiCHO P medium (GE Healthcare) supplemented with 8 mM L-glutamine and phenol red in 125 mL shake flasks (Corning), at a working volume of 45 mL and a seeding cell density of 0.3 × 10^6^ cells × mL^−1^ and a target stop criterion of culture viabilities ≤ 80%. Addition of feed media, ActiCHO Feed A and ActiCHO Feed B (both GE Healthcare) started on day four based on a glucose-controlled feeding regimen. Feed A was added in order to meet a target glucose concentration of 6.5 g × L^−1^. Feed B was added as a constant 0.28% (v/v) feed based on the actual culture volume. Sampling of fed-batch cultures for monitoring of cell concentrations and culture viability, as well as HIV Env trimer concentration and levels of glucose, lactate, ammonium, L-glutamine and L-glutamate were performed on the day of seeding, and continued on day 4 until the end of the process (culture viability ≤ 80%). Further, ConM and ConS lead clones were monitored for their ability to maintain cell-specific growth rates [µ] and specific productivities [q_p_] of trimer expression over a period of ≥70 population doublings (PDL) under routine cultivation conditions. Following small-scale fed-batch evaluation, stability monitoring, and selection of the final ConM and ConS lead clones to enter the GMP pipeline, master cell banks (MCBs) were prepared and cryo-preserved in liquid nitrogen under GMP conditions.

### Scale-up of ConM and ConS expressing CHO lead clones for GMP manufacture

Scale-up and process development for trimer production in fed-batch mode was performed in a ReadyToProcess WAVE 25 bioreactor system using ActiCHO P medium (GE Healthcare) and the respective feed media ActiCHO Feed A and ActiCHO Feed B (both GE Healthcare). Inoculation cultures were obtained by expansion of ConM and ConS lead clones from MCBs in production medium (ActiCHO P medium supplemented with 8 mM L-glutamine) in 1000 mL shake flasks (Corning) in a shaking incubator (Kuhner) (125 rpm, 37 °C, 5% CO2, 80% relative humidity) to support a starting cell density of 0.3 × 10^6^ cells × mL^−1^ in a starting volume of 8 L. WAVE 25 bioreactor runs were operated at 37 °C, dissolved oxygen was set to 30%, with no additional pH control by base addition. Sampling of fed-batch cultures was performed in 24 h intervals for monitoring of cell concentration, cell viability, levels of glucose, lactate, ammonium, l-glutamine and l-glutamate. Additionally, cell-free supernatant was collected to measure product concentration by PGT145 ELISA. Feeding of WAVE fed-batch cultures was performed as follows. Feed media addition of ActiCHO Feed A and ActiCHO Feed B (both GE Healthcare) was started once the glucose levels fell below 4 g x L^−1^. Thereafter, feeding with nutrient feeds was continued on a daily basis. In accordance with small-scale fed-batch experiments, glucose and nutrient levels were replenished by adding the respective amount of ActiCHO Feed A to a glucose target level of 6.5 g x L^−1^. ActiCHO Feed B was added as a culture volume based 0.28% (v/v) volume feed. At harvest, cells were removed by centrifugation and cell-free supernatants were passed through a positively charged 3 M™Zeta Plus™ depth filter (3 M) for removal of negatively-charged contaminants. As a final step, depth filtered supernatants were sterile filtered through a Sartopore 2 (Sartorius) filter unit and stored until further processing at 2–8 °C.

### GMP production of ConM and ConS Env trimers in fed-batch mode

GMP manufacture of ConM and ConS Env trimers was based on previously established master cell banks of ConM and ConS expressing lead clones and cultivation in fed-batch mode using a ReadyToProcess WAVE50 Bioreactor system (GE Healthcare) and purification of well-folded Env trimers from harvested culture supernatant by PGT145 affinity chromatography and subsequent polishing steps. Briefly, inoculum cultures were prepared from cryo-preserved master cell banks and cultures were expanded in production medium (ActiCHO P medium, supplemented with 8 mM L-glutamine) to support starting cell densities of ≥ 3 × 10^5^ cells x mL^−1^ at a starting volume of 17 L. WAVE GMP fed-batch production runs were operated at 37 °C, pH was set to 7.0 (controlled via base addition and purging with CO_2_), 20 rpm rocking speed (6° angle) and 30% dissolved oxygen (DO). Daily sampling was performed for analysis of cell concentration, viability, ConM and ConS Env concentration, glucose levels and key metabolites. Addition of nutrient feeds ActiCHO Feed A and ActiCHO Feed B (both GE Healthcare) started when glucose levels dropped below ≤ 4 g x L^−1^ and was continued daily thereafter. ActiCHO Feed A was added to a final glucose concentration of 6.5 g x L^−1^, ActiCHO Feed B was added as a 0.28% (v/v) volume feed based on the working volume in the bioreactor. Once viabilities dropped below 80% ( ≥ 60%) fed-batch cultures were terminated and culture supernatants were harvested by 2-stage depth filtration using 3 M™ ZetaPlus™ filters for cell removal, followed by removal of host cell proteins (HCP) and DNA by anion-exchange (AEX) membrane absorption using 3 M™ Emphaze™ Hybrid Purifier filters, followed by a final 0.2 µm sterile filtration step using Polyethersulfone (PES) filters (PALL).

### Purification of ConM and ConS Env trimers

Fed-batch supernatants were concentrated ( ≤ 14-times) and dia-filtered ( ≥ 6-times buffer exchange) into 20 mM Tris / 500 mM NaCl / pH 8.3 using Sartocon ECO (30 kDa MWCO, 0.70 m²) PES membranes (Sartorius). Buffer exchanged supernatants were filtered using 0.8 + 0.45 µm Sartopore 2 filters (Sartorius) for particulate contaminant removal and further sterile filtered using 0.2 µm Sartopore 2 filters (Sartorius), then divided into four parts for further processing. Virus inactivation was performed by addition of 1% (w/w) Triton X-100 and incubation at RT for 60–120 mins and solutions sterile filtered using 0.45 µm Sartopore 2 filters (Sartorius). Triton X-100 inactivated solutions were batch-wise loaded onto a MAb PGT145 immunoaffinity column for capture of ConM and ConS Env trimers. The affinity columns were prepared from an in-house prepared GMP stock of MAb PGT145 by coupling of the antibody to Toyopearl 650 M chromatography resin (Tosoh). Loaded columns were washed with ≥ 10 column volumes of 20 mM Tris / 500 mM NaCl / pH 8.3 and bound ConM and ConS trimers were eluted from the columns with 50 mM histidine / 2 M MgCl_2_ / pH 6.0. Trimer elution fractions were concentrated ( ≤ 5-times) and dia-filtered ( ≥ 10-times buffer exchange) to 0.1 M Glycine / 10 mM Tris / pH 7.5 using a Sartocon Slice ECO (30 kDa MWCO, 0.14 m²) PES membrane (Sartorius). Trimer-containing fractions were pooled and passed over a pre-equilibrated MAbSelect SuRe Protein A (ProA) affinity column (GE Healthcare) to remove leached residual PGT145-affinity ligand from the previous column. The trimer containing ProA flow through fractions were loaded onto a Q-Sepharose FF anion-exchange column (GE Healthcare), and columns washed with ≥ 5 column volumes of 0.1 M Glycine / 0.01 M Tris / pH 7.5. Trimers were then actively eluted with 0.1 M Glycine / 0.01 M Tris / 0.25 M NaCl / pH 7.5 (injection grade). AEX eluate fractions were sterile filtered using 0.45 + 0.2 µm Sartobran 300 filters (Sartorius), concentrated and dia-filtered to 20 mM Tris / 150 mM NaCl / pH 7.5 (injection grade drug substance/product buffer) using a Sartocon Slice ECO (30 kDa MWCO, 0.14 m²), diluted to a final Env trimer concentration of 2 mg/mL and filtered using 0.45 + 0.2 µm Sartobran 300 filter capsules (Sartorius). As final steps, nano-filtration of formulated ConM and ConS drug substance batches was performed using Planova™ 20 N (0.3 m²) (Asahi Kasei) filters for virus removal, and drug substance batches were further diluted to concentrations of ~1 mg/mL in drug substance/drug product buffer (20 mM Tris / 150 mM NaCl / pH 7.5) and stored for further processing at 2–8 °C.

### EDC cross-linking of GMP produced trimers and PGT145 immunoaffinity purification

ConM and ConS trimer preparations manufactured under GMP conditions were cross-linked by EDC / sulfo-NHS chemistry. As a preparatory step, ConM and ConS bulk drug substance (DS) (1 mg x mL^−1^; 20 mM Tris / 150 mM NaCl / pH 7.5; injection grade) were concentrated and dia-filtered into their optimal cross-linking buffer environments. ConM bulk DS preparations were buffer exchanged to 20 mM HEPES / 150 mM NaCl / pH 7.5; injection grade, and ConS DS preparations were buffer exchanged to phosphate buffered saline (PBS), pH 7.4; injection grade ( ≥ 10 volume changes) using a Sartocon Slice ECO (30 kDa MWCO, 0.14 m²) PES membrane (Sartorius). Buffer exchanged DS preparations were further adjusted to a concentration of ~1 mg x mL^−1^ in 20 mM Tris / 150 mM NaCl / pH 7.5; injection grade. EDC cross-linking conditions were different for ConM and ConS trimers. For both trimers, EDC and NHS stock solutions were diluted in the trimer specific buffers and mixed with equal amounts (w/w) of buffer exchanged trimer solution (1 mg x mL^−1^). Cross-linking reactions for ConM were performed at RT for 30 mins, and ConS was incubated for 10 mins. Optimal molar concentrations for cross-linking of ConM and ConS were 1 M EDC / 10 mM NHS and 0.25 M EDC / 10 mM NHS respectively. Cross-linking reactions were quenched by addition of equal amounts (w/w) of a 1 M Glycine, pH 7.4 stock solution and incubation at RT for ≥ 10 mins. Further, cross-linked ConM (ConM-EDC) and ConS (ConS-EDC) preparations were concentrated and dia-filtered ( ≥ 7-times buffer exchange) with 20 mM Tris / 500 mM NaCl / pH 8.3; injection grade and passed through a 0.45 + 0.2 µm Sartopore 2 300 (Sartorius) filter for sterile filtration. Processed ConM-EDC and ConS-EDC trimer preparations were loaded onto an in-house prepared GMP grade MAb PGT145 immunoaffinity column (MAb PGT145 coupled to Toyopearl 650 M chromatography resin by Tosoh) for positive selection of well-folded, ConM-EDC and ConS-EDC trimers. The loaded affinity column was washed with ≥ 3 column volumes of 20 mM Tris / 500 mM NaCl / pH 8.3 (injection grade) and ConM-EDC / ConS-EDC trimers were eluted from the column with elution buffer (50 mM histidine / 2 M MgCl2 / pH 6.0; injection grade). Elution ( ≥ 1 column volume) was controlled by in-line monitoring of the absorbance signal (AU/cm) at 280 nm. Immediately after, ConM-EDC and ConS-EDC MAb PGT145 immunoaffinity eluates were concentrated ( ≤ 5-times) and dia-filtered ( ≥ 14-times buffer exchange) into drug substance (DS) formulation buffer 20 mM Tris / 150 mM NaCl / pH 7.5; injection grade. As a last step, formulated ConM-EDC and ConS-EDC solutions were 0.2 µm filtered using a PES beaker filter (Millipore), concentrations were adjusted to ~1 mg x mL^−1^ and the final DS preparations were stored at 2–8 °C.

### PGT145 ELISA for quantification of ConM and ConS from supernatant samples

96-well plates (Nunc, Maxisorp) were coated with 1 µg x mL^−1^ of MAb 2G12 (Polymun Scientific) in carbonate/bicarbonate coating buffer, pH 9.5 at 4 °C overnight. Before use, plates were blocked with PBS + 0.1% Tween and 1% BSA (PBS-T, 1% BSA) at RT for 1 h. Supernatant samples and reference standards were diluted performing a 1:2 serial dilution series in PBS-T, 1% BSA. Assay plates (pre-coated) were washed (4-times) with PBS-T and aliquots of serially-diluted samples and standards were transferred to pre-coated assay plates and incubated at RT for 1 h. After washing (4-times with PBS-T), biotinylated MAb PGT145 at 1 µg/mL was applied for 1 h. Following incubation with MAb PGT145, plates were washed and incubated with streptavidin-HRP conjugate (Roche) for 30 mins. Color development was started by addition of o-phenylenediamine dihydrochloride (OPD) substrate, the reaction stopped by addition of 25% H_2_SO_4_ (Merck) and OD492 was measured with a Synergy 2 plate reader (BioTek). Evaluation of unknown supernatant samples by interpolation from standard curves of ConM and ConS reference material was performed using the Gen5 Microplate Reader and Imager Software package, version 3.05 (BioTek).

### Characterization of GMP produced ConM, ConM-EDC, ConS and ConS-EDC trimer preparations

Trimer integrity and stability of GMP produced ConM and ConS trimer preparations and their EDC cross-linked versions were subject to long-term stability monitoring of up to 60 months. Trimer preparations were evaluated by BN PAGE (Native PAGE Novex 3–12% Bis-Tris Gels) and Size Exclusion HPLC (SE-HPLC) using 2 consecutive Acquity UPLC protein columns (Waters) with 200 Å and 450 Å to asses integrity and purity of GMP trimer preparations. Reducing and non-reducing SDS PAGE (NuPAGE 4 to 12%, Bis-Tris gels) was performed together with immunoblotting and probing with anti-Env specific antibodies (5F3, 447–52D, 2G12). GMP-produced preparations were routinely monitored for the presence of particulate contaminants, pH, endotoxin levels and osmolality. The SDS-PAGE characterization shown in Fig. 1c was carried out in one experiment and all samples were processed in parallel.

### DSC analysis

Thermal denaturation of unmodified and cross-linked GMP batches of ConM and ConS was studied using a nano-DSC calorimeter (TA instruments, Etten-Leur, The Netherlands)^33^. Briefly, trimers were first dialyzed against PBS and concentration adjusted to ~0.25 or ~1.0 mg/mL, respectively. After sample loading, thermal denaturation was probed at a scan rate of 60 °C/h. Buffer correction, normalization, and baseline subtraction procedures were applied and data were analyzed using the NanoAnalyze Software v.3.3.0 (TA Instruments). The data were fitted using a non-two-state model, as the asymmetry of some of the peaks suggested that unfolding intermediates were present. DSC experiments were performed with a D7324-tagged trimer, but the presence of the D7324-tag did not alter the *T*_m_ values compared to the corresponding non-tagged trimers^33^.

### Free amine assay

Unmodified or cross-linked trimer (5 μg) in 20 μL PBS was added to 30 μL of 0.1 M NaHCO_3,_ pH 8.5. 25 μL of 5% 2,4,6-Trinitrobenzene Sulfonic Acid (TNBSA) diluted 1/500 in 0.1 M NaHCO_3_ pH 8.5 was added to the samples for 2 h at 37 °C, followed by 25 μL of 10% SDS and 12.5 μL of 1 M HCl. Samples were vortexed and the optical density read at 335 nm. The relative quantity of free amines was calculated as (OD_335_ (GLA-SOSIP trimer)–OD_335_ (blank)) / (OD_335_ (SOSIP trimer)–OD_335_ (blank)).

### Capture ELISA for Env trimer binding by human mAbs

ELISA plates (Greiner Bio-One) were coated with 4 μg/mL of capture mAb 2G12 at 4 °C overnight in PBS. After blocking with 2% BSA/PBS + 0.05% Tween, trimer (0.2 μg/mL) were captured, labeled with a titration series of biotinylated human mAbs followed by peroxidase-conjugated streptavidin detection reagent (Jackson ImmunoResearch). The colorimetric endpoint was obtained using the one-step ultra TMB substrate (Thermofisher). MAbs were developed until a signal of ~1–2 optical density 450 nm (OD_450_) units was generated for each antibody, leading to longer incubation periods for non-Nabs, and color development was stopped with sulfuric acid (0.5 M), and the OD_450–570_ measured. All ELISA signals were corrected by subtracting the background signal obtained in the absence of primary antibody and the resulting data were plotted against the log_10_ of the antibody concentration using GraphPad Prism V7.0. To generate binding indices from ELISA titration curves, an area under the curve (AUC) analysis of ligand-trimer binding was performed; the binding index represents the ratio of the cross-linked trimer value to the value of the matched unmodified SOSIP trimer that was used for cross-linking. Binding indices were calculated as (AUC(GLA-SOSIP trimer)—AUC(blank))/(AUC(SOSIP trimer)—AUC(blank)), where blank = negative control curve of the respective mAb without antigen. Indices <1 indicate reduced binding to the cross-linked trimer compared to its unmodified counterpart, and the converse for values > 1.

### Env trimer ELISA for mouse sera

Detection of mouse trimer-specific serum antibodies was performed using endpoint titer ELISA. 2G12 antibody (4 μg/mL, 50 μL/well) was captured overnight at 4 °C onto high-protein-binding ELISA plates (Spectraplate 96HB, Perkin Elmer). Plates were washed in PBS/Tween (0.05% v/v) and wells blocked using BSA (2% w/v; 200 μL/well) for 1 h at RT, and washed. An unmodified trimer (0.2 μg/mL, 50 μL/well) was added for 2 h at RT and washed as before. Mouse serum samples diluted in PBS/BSA (1% w/v) starting at 1:100 then stepwise 5-fold were added to the ELISA plates (50 μL/well) and incubated overnight at 4 °C. Plates were washed and Peroxidase-conjugated rabbit anti-mouse IgG antibody (1:5000; 50 μL/well, Jackson Immunoresearch) added to all wells for 1 hr at RT. Plates were washed and TMB substrate (50 μL/well, Thermofisher Scientific) was added to all wells. Color development was monitored and terminated after 10 mins using sulfuric acid (0.5 M; 50 μL/well). Optical density (OD) values for each well were calculated as OD_450–570nm_. Background values (OD_no serum_) were subtracted from sample readings. Endpoint titers were calculated using non-linear regression curve fitting and interpolated values transformed into log_10_ endpoint titers (Graphpad Prism 7 for Mac).

### Biolayer interferometry (BLI) trimer antigenicity analysis

The GMP trimer constructs in this study are devoid of tags, therefore Fab fragments were loaded onto anti-Human Fab CHI (FAB2G) biosensors and dipped into varying concentrations (in nM: 2000, 1000, 500, 250, 125, 62.5, 31.25) of trimer using an OctetRed 96 instrument (ForteBio). Trimers and Fabs were diluted into 1× kinetics buffer (PBS pH 7.4 + 0.01% BSA, 0.002% Tween-20), and allowed to reach room temperature before beginning the assay. Association was measured for 600 seconds, followed by dissociation for 1200 seconds in 1× kinetics buffer. A reference well-containing kinetics buffer was subtracted from each data set, curves were aligned on the *y* axis using the baseline step, and an inter-step correction was applied between the association and the dissociation curves. A 1:1 binding model, which assumes first-order kinetics and each binding site on the SOSIP binds to immobilized Fab at an equal rate, was fitted to the data and used to determine kinetic parameters.

### Analysis of elastase and Cathepsin G activity on trimers

To test Env stability in the presence of neutrophil-released proteases, 0.01 Units of elastase or cathepsin G (Sigma) was added to 1 μg of each trimer in a final volume of 11.1 μL PBS and incubated at 37 °C for 1 or 24 h. A negative control, containing no enzyme, also underwent a 24 h incubation period at 37 °C. After protease exposure, the samples were analyzed by reducing and denaturing SDS-PAGE on 4–12% bis-tris gels (Life Technologies). Protein bands were developed with Simply Blue Safe Stain (Life Technologies) and band intensity was analyzed on Biorad GelDoc XR with Lab Image software. The SDS-PAGE gel in Fig. 1e was derived from one representative experiment (original SDS-PAGE gels shown in Supplementary Fig. 3) and all samples were processed in parallel. Band intensity of the untreated control was set at 100% and all other samples were normalized to that.

### TZM-bl neutralization assay

IMC ConM and ConS^4^ were produced in HEK293T cells, titered, and used in a TZM-bl assay^53^ to determine nAb responses. Duplicates of six steps of threefold dilution, starting with 1:20 of each serum, were incubated with viral supernatant (at relative luminescence units (RLU) between 150,000 and 200,000) for 1 h. Thereafter, 10^4^ TZM-bl cells were added, and plates were incubated for 48 h at 37 °C, after which Bright-Glo Luciferase assay system (Promega, Madison, Wisconsin, USA) was added to measure luciferase activity with a Mithras luminometer (Berthold, Germany). Positive controls were sera of HIV-1-infected individuals and monoclonal antibodies with known neutralizing titers. Neutralization titers were defined as the sample dilution at which RLU was reduced by 50% compared to virus control wells after subtraction of background RLU in control wells with only cells. Inhibitory concentrations (IC) 50 were calculated with a linear interpolation method using the mean of the duplicate responses^53^.

### Neutralization depletion experiment

To interrogate the V1V2 specificity of the rabbit antiserum nAb responses against the ConM IMC, sera were first incubated for 1 h at room temperature with 40 µg/mL of the soluble ConM SOSIP.v7 trimer with or without swapped V1V2 loops, constructed by replacing the V1V2 of ConM (residues 131–196 in HXB2 numbering) with that of BG505^35^. All ConM SOSIP.v7 depletion reagents contain the D368R mutation to abrogate binding to CD4 on the TZM-bl cell line and were positively selected by PGT145 affinity chromatography to ensure that only native-like trimers were used in the depletion experiments. Subsequent steps of the neutralization assay were performed as described above.

### T-cell cytokine responses

Spleens were harvested from mice 4 weeks after the final boost, dissected using an aseptic technique, and single cells isolated by passing through a 100 μm filter. Splenocytes were resuspended in supplemented RPMI (10% fetal bovine serum, 10 mM HEPES, 2 mM Glutamax, 1× Penicillin–Streptomycin, 50 μM 2-ME) and plated at 500,000 splenocytes per well in 96 U-bottomed plates in a final volume of 200 μL. Splenocytes were either pulsed with relevant antigen at 50 μg/mL or no antigen as a control. On day 3, 100 μL supernatant was harvested and frozen at −80 °C for cytokine analysis. The concentration of IL-2 in 2× diluted supernatants was measured using an IL-2 mouse ELISA kit (Thermo Fisher Scientific) as per the manufacturer’s instructions. The concentration of IL-2, IFNγ, IL-5, IL-13, and IL-17a in supernatants was measured using a Luminex multiplex assay (R&D) performed according to the manufacturer’s instructions. Briefly, supernatant was diluted 1:2 in RD1W buffer, whilst the standards were prepared as instructed. 50 μL of standard or diluted supernatant was pipetted into the wells of a black 96-well plate and 50 μL of magnetic-bead cocktail was added to each well. The plates were then incubated for 2 h on a shaker set to 800 rpm. The supernatant was removed and wells were washed 3× with 300 μL of wash buffer using a magnetic plate washer. 50 μL of biotin-labeled secondary antibody, diluted as per the instructions was added to the wells and the plate returned to the shaker for 1 h. The plates were washed again before a 30 min incubation with streptavidin-PE, washed and the plates read on a Luminex Bio-Plex (Bio-Rad).

### Negative stain EM

GMP SOSIPs were diluted in TBS pH 7.4 to ~0.02 mg/mL, applied to glow-discharged copper EM grids containing a continuous carbon film, then negatively stained with 2% uranyl formate for ~10 s. Micrographs were collected on a Talos 200 C transmission electron microscope (ThermoScientific) operated at 200 keV, at a nominal magnification of ×73,000 resulting in a pixel size of 1.98 Å. CTF estimation was performed with CTFFIND4^54^, and then all subsequent processing steps were carried out in RELION-3^55^. Particles were picked with the RELION Gaussian picker, and an initial 2D classification was performed to eliminate noise and non-protein particle picks. A second round of classification was then used to determine the percent of trimeric and properly folded SOSIPs within each GMP sample. The percent well-folded trimers were reported as the number of particles contained within 2D classes of trimeric SOSIPs, divided by the total number of particles in the particle stack.

### Cryo-electron microscopy sample preparation

Unmodified and cross-linked GMP SOSIP trimers were incubated with a sixfold molar excess of PGT122 Fab overnight at 4 °C. Excess Fab was removed by ultrafiltration in TBS pH 7.4 with a 100 kDa cutoff Centricon filter (Amicon Ultra, Millipore), and the sample was concentrated to ~5 mg/mL for cryo grid freezing. Immediately before vitrification, *n*-Dodecyl β-d-maltoside was added to a final concentration of 0.25 mM, which improved the angular distribution of the protein particles in vitreous ice. 3 µL of this mixture was applied to Quantifoil R1.2/1.3 holey carbon Cu 400 mesh grids, and blotted and plunge-frozen in liquid ethane with the Vitrobot Mark IV.

### Cryo-electron microscopy data collection

Micrographs were collected on a Titan Krios (ThermoScientific) operating at 300 keV coupled with a Gatan K2 direct electron detector via the Leginon interface^56^. Each exposure image was collected in counting mode at ×29,000 nominal magnification resulting in a pixel size of 1.03 Å/pixel, using a dose rate of ~5.5 e-/pix/sec, and 200 ms exposure per frame. The total dose on the sample for each movie micrograph was 50 e-/Å^2^, and the nominal defocus range used was −1.0 to −2.2 μm. These imaging conditions were kept consistent for each of the four samples (ConS, ConS-EDC, ConM, and ConM-EDC).

### Cryo-electron microscopy data processing

Movie micrograph frames were aligned and dose-weighted using MotionCorr2^57^, and imported into cryoSPARC v2^58^. CTF models were calculated using CTFFIND4. The cryoSPARC blob picker was used on a subset of micrographs to generate an initial set of particle picks, which were subjected to 3 rounds of 2D classification. High-quality and representative classes were selected and used as 2D templates for template picking on the entire data set. The resulting particle images were subjected to multiple rounds of 2D classification, followed by one round of ab initio reconstruction to generate an initial model. This map was used as an initial model for one round of heterogeneous refinement, specifying two or four classes. For each data set, there was little observable heterogeneity, but this step served to further eliminate low-resolution particles. Particles from the dominant class(es) were re-extracted with a box size of 352 pixels, and subjected to homogeneous refinement. Next, iterative rounds of global CTF refinement followed by homogenous refinement were performed until the resolution had converged, followed by a final round of non-uniform refinement. The ConS data showed the potential to reach a higher resolution, therefore these data were first processed in RELION according to standard single-particle workflows in order to perform Bayesian polishing. This map reached a resolution of 3.3 Å, but PGT122 Fab density was poorly resolved. Polished particles were extracted and imported into cryoSPARC v2, and the same workflow as described above was performed for this particle stack. This procedure led to a very high-quality reconstruction throughout the complex, with a final resolution of 3.1 Å. For unmodified ConM, higher resolution was achieved by importing frames into cryoSPARC2 and using the native patch motion correction utility. This allowed for local (single particle) motion to be corrected, which improved the resolution and led to the final 3.4 Å reconstruction. For EDC cross-linked ConS and ConM, these procedures led to no further improvements, thus for these data, pre-aligned and dose-weighted micrographs were imported, as described above. All resolutions are reported according to the “gold standard” 0.143 FSC cutoff^59^.

### Model building

The initial model for ConM-PGT122 comprised the ConM SOSIP coordinates from the ConM-PGT122-35022 crystal structure (PDB 6IEQ), and the PGT122 Fab coordinates from a crystal structure of BG505-PGT122-35022 (PDB 4TVP). These coordinates served as the template for complete rebuilding and all-atom refinement with RosettaCM^60^, which couples the fragment-based de novo Rosetta builder with sequence and structural constraints from the template(s), as well as a user-defined electron density weight. The lowest energy model was selected and glycans were added to the model in Coot^61^, which was then iteratively refined in real-space with Coot and PHENIX^62^. Once the model had converged, disordered regions were removed and a final high-resolution, all-atom refinement with Rosetta Relax^63^ was performed. This ConM-PGT122 cryo-EM structure then served as the initial model for both ConM-EDC and unmodified ConS, which followed a nearly identical procedure: full model rebuilding with RosettaCM, real-space refinement in Coot and PHENIX, and final refinement with Rosetta Relax. This same procedure was also used for the ConS-EDC structure, except that the unmodified ConS structure was used as the initial model. Structure validation was performed with MolProbity^64^, PHENIX, and EMRinger^65^.

### RMSD analysis

Alpha carbon (Cα) root-mean-square deviation (RMSD) was calculated by superimposition of structures in UCSF Chimera^66^. This was performed globally and locally, in order to differentiate global conformational changes from local changes in structure of specific epitopes. Values for global RMSD are listed in Fig. 2b–e, and Supplementary Fig. 9. The local Cα RMSD was mapped onto the cryo-EM structures in Fig. 2b–e using UCSF Chimera.

### Mouse immunizations

All experiments used 8–12 week-old female BALB/c mice (Charles River) maintained under specific pathogen-free conditions in the Sir William Dunn school facility. In two independent experiments, 4 or 5 mice per group were immunized by subcutaneous administration with 10 μg trimer formulated in 100 μL PBS/MPLA (10 µg/dose) formulation on weeks 0, 4, and 8. Mice were monitored for adverse symptoms throughout. Blood was collected by a tail bleed on weeks −1, 2, 6, 10, and 12, and serum was separated and stored at −20 °C.

### Rabbit immunizations, IgG purification, and ELISA

A total of 30 New Zealand White Specific Pathogen-Free (SPF) rabbits (23 males and 23 females (were nulliparous and non-pregnant)), 11 weeks old and weighing approximately 2 kg, were immunized intramuscularly at the Research Toxicology Centre S.p.A, Italy. Each study group (detailed in Supplementary Fig. 13B) consisted of three male and three female animals. The rabbits received a total of four administrations (Weeks 0, 3, 6, and 9) into the lateral surface of the quadriceps muscle of both legs. Rabbit serum was isolated from blood at 0, 3, 6, 9- and 12 weeks first post-vaccination, heat-inactivated, aliquoted, and stored at −20 °C prior to assessment for antigen-specific IgG. Antigen-specific gp140 binding antibodies were measured using standardized ELISA platforms. In serum samples, antigen-specific IgG was measured. In brief, 96-well medium binding plates (Griener, Kremsmunster, Austria) were coated with either recombinant ConM, ConM-EDC, ConS, or ConS-EDC gp140 (Polymun Scientific) at 1 μg/mL in PBS for 1 h 37 °C. As reference material, standard immunoglobulins (Serotec, UK) were captured with anti-Rabbit specific goat antibodies (Millipore, UK). After blocking with assay buffer (5% bovine serum albumin; Sigma–Aldrich; 0.05% Tween ThermoFisher Scientific, Pittsburgh, PA), samples were initially screened at 1:100 dilution (then titrated to optimal dilutions) on antigen-coated wells, and serial dilutions of immunoglobulin standards were added to the anti-Rabbit capture antibody coated wells and incubated for 1 h at 37 °C. Secondary antibody HRP-conjugated anti-rabbit IgG was added at 1:20,000 dilution and incubated for 1 h at 37 °C. Plates were developed with SureBlue TMB substrate (KPL, Insight Biotechnology, London, United Kingdom). The reaction was stopped after 5 min by adding TMB stop solution (KPL, Insight Biotechnology), and the absorbance was read at 450 nm on a VersaMax 96-well microplate reader (Molecular Devices, Sunnyvale, CA). The ELISA data are expressed as positive if the blank subtracted OD450 nm was above the predetermined cutoff of OD 0.2 nm and values are on the linear range of the curve. To ensure assay sensitivity, a positive control composed of gp140-positive pooled rabbit serum samples and a negative control composed of anti-Ad4 Rabbit Hypersera (PAXVAX, San Diego, US) were used. Analyses of the data were performed using SoftMax Pro GxP software v6.5 (Molecular Devices).

### Ethics statement

Animal research using rabbits and mice was carried out in full accordance with local and national ethical guidelines. All protocols for breeding and procedures with mice were approved by the Home Office UK, under the Animals (Scientific Procedures) Act 1986 and Home Office license PPL3003421. Rabbit studies were carried out at Covance Inc.

### Statistical analyses

Statistical analysis was performed in Prism using the tests described in the corresponding figure legends. Briefly, one-way ANOVA of log-transformed data with Sidak’s post-test correction to account for multiple comparisons was used to analyze normally distributed data including log-transformed endpoint titers. Non-parametric analysis (not assuming a Gaussian distribution) between two independent groups was performed using a two-tailed Mann—Whitney *U* test and an unmatched, unpaired Kruskal–Wallis test with Dunn’s multiple comparison test was used to compare non-normally distributed data with more than one comparison. All error bars represent ±1 SD.

### Reporting summary

Further information on research design is available in the Nature Research Reporting Summary linked to this article.

## Supplementary information

Supplemental Information

REPORTING SUMMARY

## Data Availability

The final cryo-EM reconstructions and the resulting structural models have been deposited into the Protein Data Bank (PDB) the Electron Microscopy Data Bank (EMDB) under the following accession codes: ConS: PDB 7LX2, EMDB 23564; ConS-EDC: PDB 7LX3, EMDB 23565; ConM: PDB 7LXM, EMDB 23571; ConM-EDC: PDB 7LXN, EMDB 23572.
