# Supplementary material for: Profound structural conservation of chemically cross-linked HIV-1 envelope glycoprotein experimental vaccine antigens
Source: NPJ Vaccines. 2023 Jul 13;8:101. doi: 10.1038/s41541-023-00696-w (PMC10345191; doi:10.1038/s41541-023-00696-w)
Supplement: Supplementary file 1 — Supplemental Information [file 41541_2023_696_MOESM1_ESM.pdf]

Supplemental Information for

Profound structural conservation of chemically cross-linked HIV-1 envelope glycoprotein experimental vaccine antigens

Gregory M Martin[1,10*], Rebecca A Russell[2,10], Philip Mundsperger[3,4], Scarlett Harris[2], Lu Jovanoska[2], Luiza Farache Trajano[2], Torben Schiffner[2,5], Katalin Fabian[6], Monica Tolazzi[7], Gabriella Scarlatti[7], Leon McFarlane[8], Hannah Cheeseman[8], Yoann Aldon[8], Edith E. Schermer[9], Marielle Breemen[9], Kwinten Sliepen[9], Dietmar Katinger[3,] Renate Kunert[4], Rogier W Sanders[9], Robin Shattock[8], Andrew B Ward[1*], Quentin J Sattentau[2*]

Supplemental File Contains

Supplementary Figures 1 - 14

Supplementary Tables 1 - 2

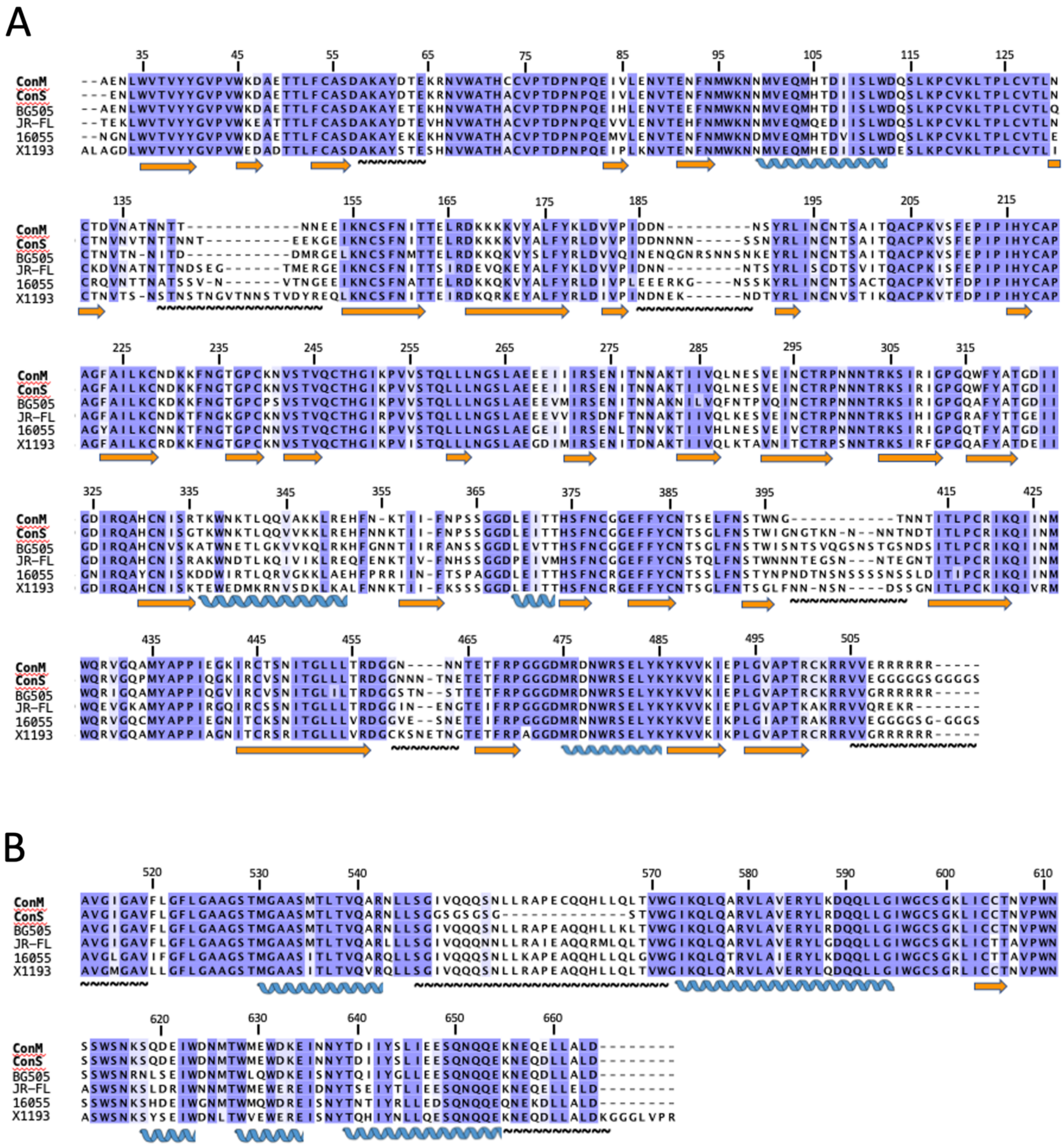

Supplementary Figure 1. Sequence alignment of ConM and ConS with Env sequences from various clades. BG505, clade A; JR-FL, clade B; 16055 NFL TD, clade C; X1193.c1 SOSIP.664, clade G. **A)** gp120 alignment, **B)** gp41 alignment. Orange arrows denote beta strands, blue coils denote alpha helices, and tilde strings are regions mostly disordered in the ConM and ConS structures. Numbering is according to the HXB2 system.

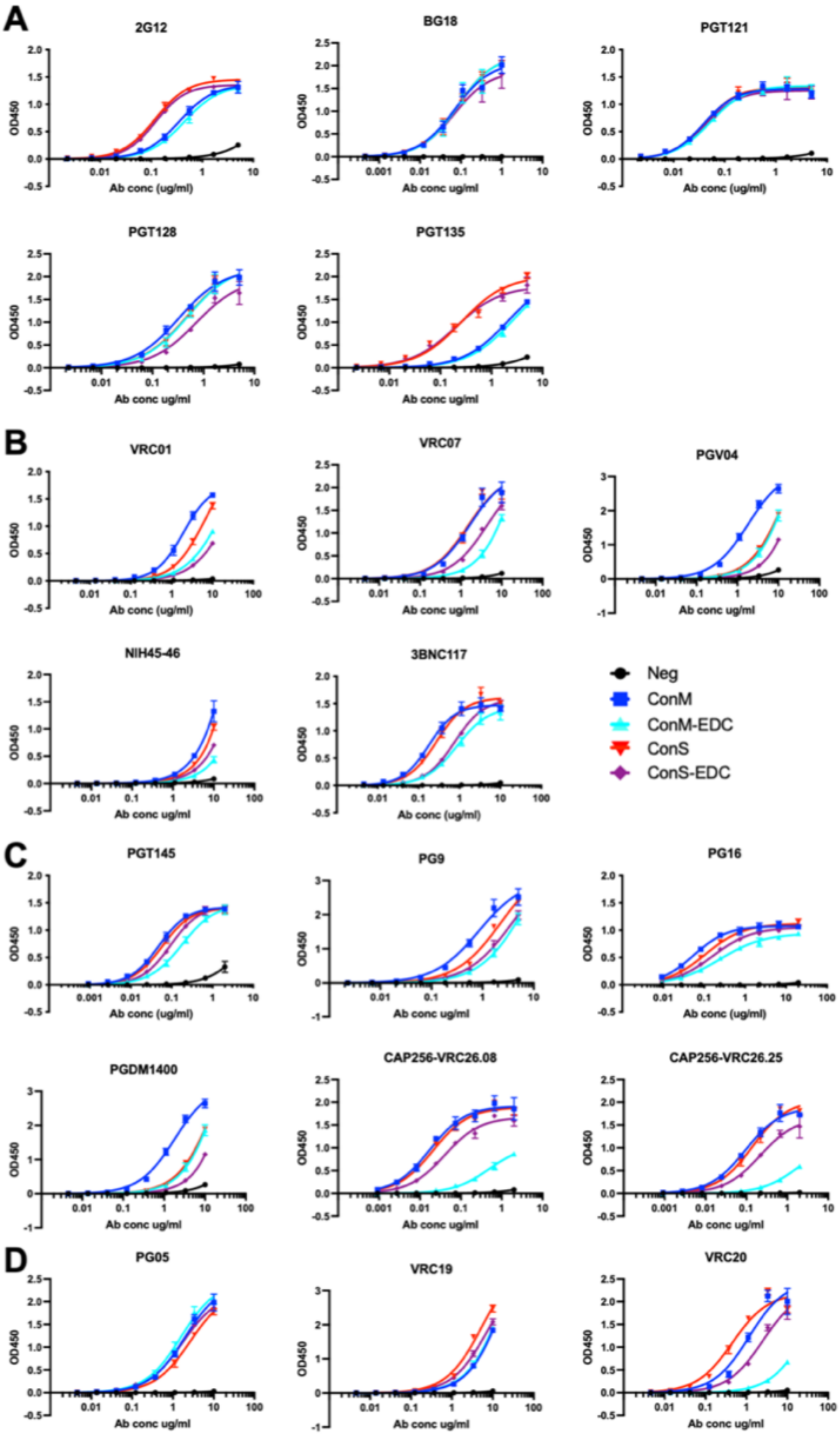

Supplementary Figure 2. ELISA binding curves associated with Figure 1G.
A) V3 glycan bnAbs; **B**) CD4 binding site bnAbs; **C**) Apex bnAbs; **D**) Silent face bnAbs

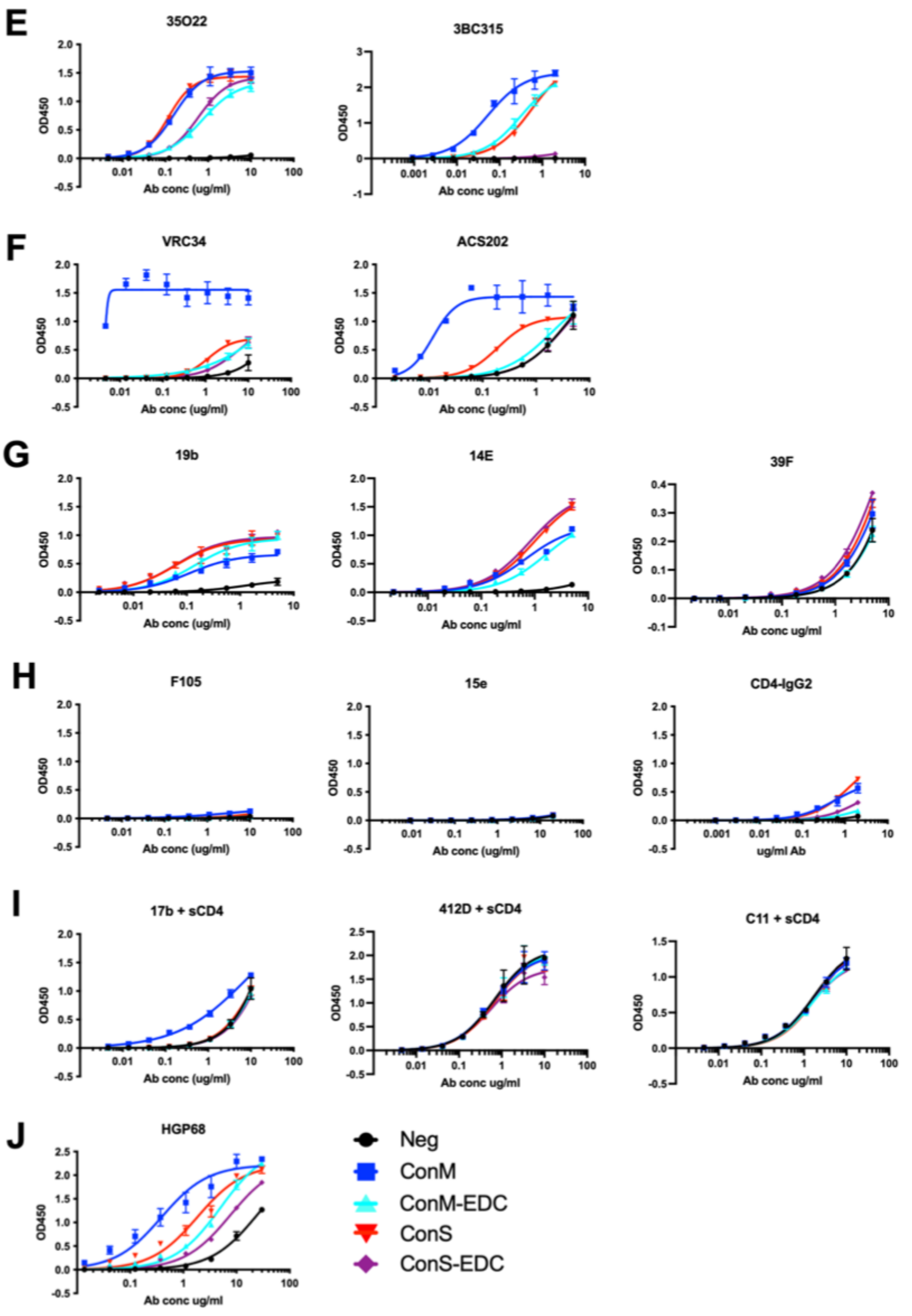

Supplementary Figure 2. ELISA binding data associated with Figure 1G.
A) V3 glycan bNAbs; **B)** CD4bs bNAbs; **C)** Apex quaternary bNAbs; **D)** Silent face bNAbs; **E)** gp120-gp41 interface bNAbs; **F)** fusion peptide bNAbs; **G)** V3 non-Nabs; **H)** CD4bs non-Nab and CD4 IgG2; **I)** CD4i non-NAb; **J)** V2 non-NAb.

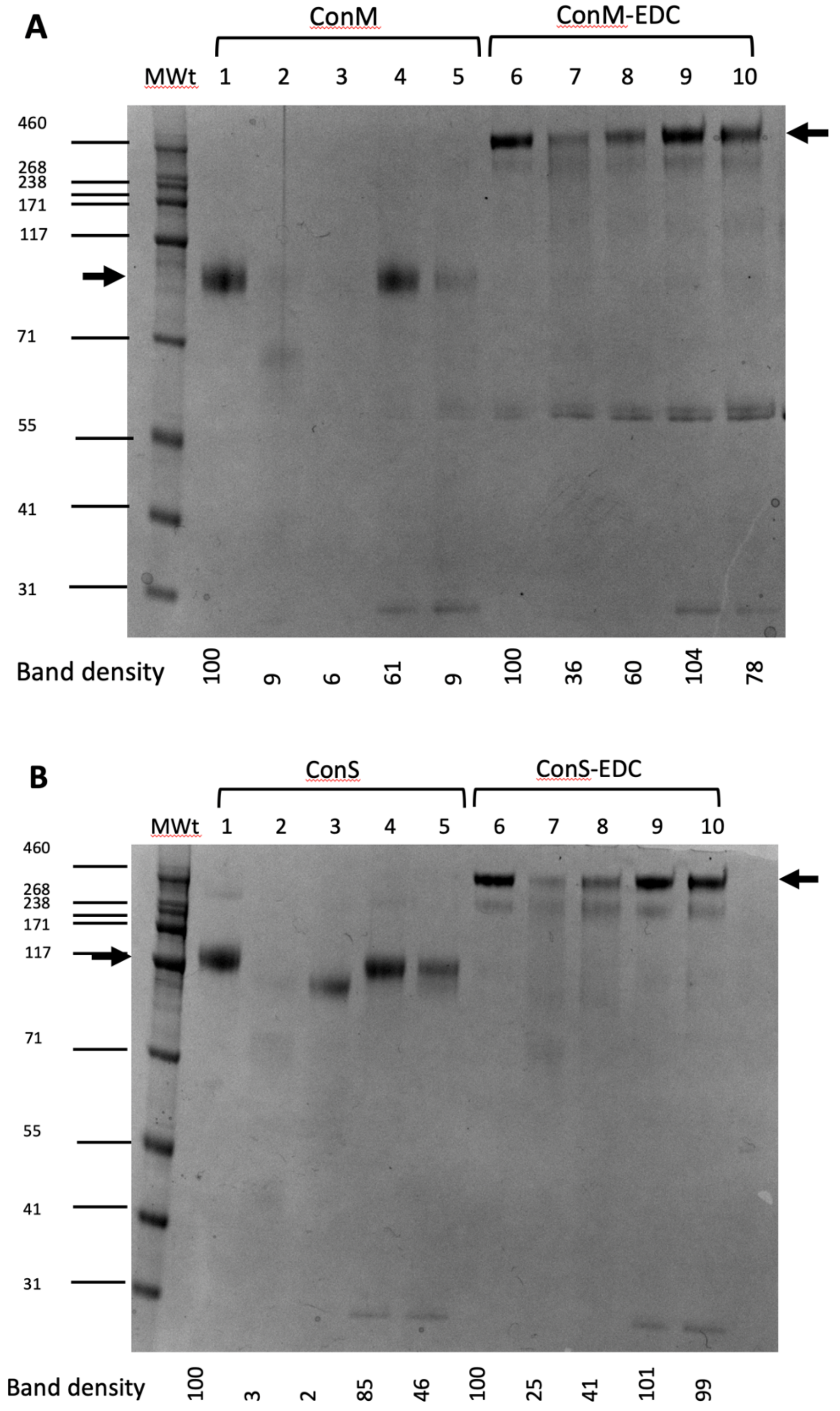

Supplementary Figure 3. Complete SDS-PAGE gels corresponding to Fig. 1E.
A) ConM and ConM-EDC treated with elastase (E) or Cathepsin G (Cg) for different times and subjected to SDS-PAGE. Lane: 1, ConM untreated, 24 h; 2, ConM + E 1 h; 3, ConM + E 24 h; 4, ConM + Cg 1 h; 5, ConM + Cg 24 h; 6, ConM-EDC untreated 24 h; 7, ConM-EDC + E 1 h, 8, ConM-EDC + E 24 h; 9, ConM-EDC + Cg 1 h; 10, ConM-EDC + Cg 24 h. Left arrow represents SDS-PAGE dissociated ConM gp140 monomer, right arrow represents SDS-PAGE ConM-EDC gp140 trimer. Numbers below SDS-PAGE gel represent densitometry readings for each lane. **B**) ConS and ConS-EDC treated with elastase (E) or Cathepsin G (Cg) for different times and subjected to SDS-PAGE. Lane: 1, ConS untreated, 24 h; 2, ConS + E 1 h; 3, ConS + E 24 h; 4, ConS + Cg 1 h; 5, ConS + Cg 24 h; 6, ConS-EDC untreated 24 h; 7, ConS-EDC + E 1 h, 8, ConS-EDC + E 24 h; 9, ConS-EDC + Cg 1 h; 10, ConS-EDC + Cg 24 h. Left arrow represents SDS-PAGE dissociated ConS gp140 protomer, right arrow represents SDS-PAGE ConS-EDC gp140 trimer. Numbers below SDS-PAGE gel represent densitometry readings for each lane.

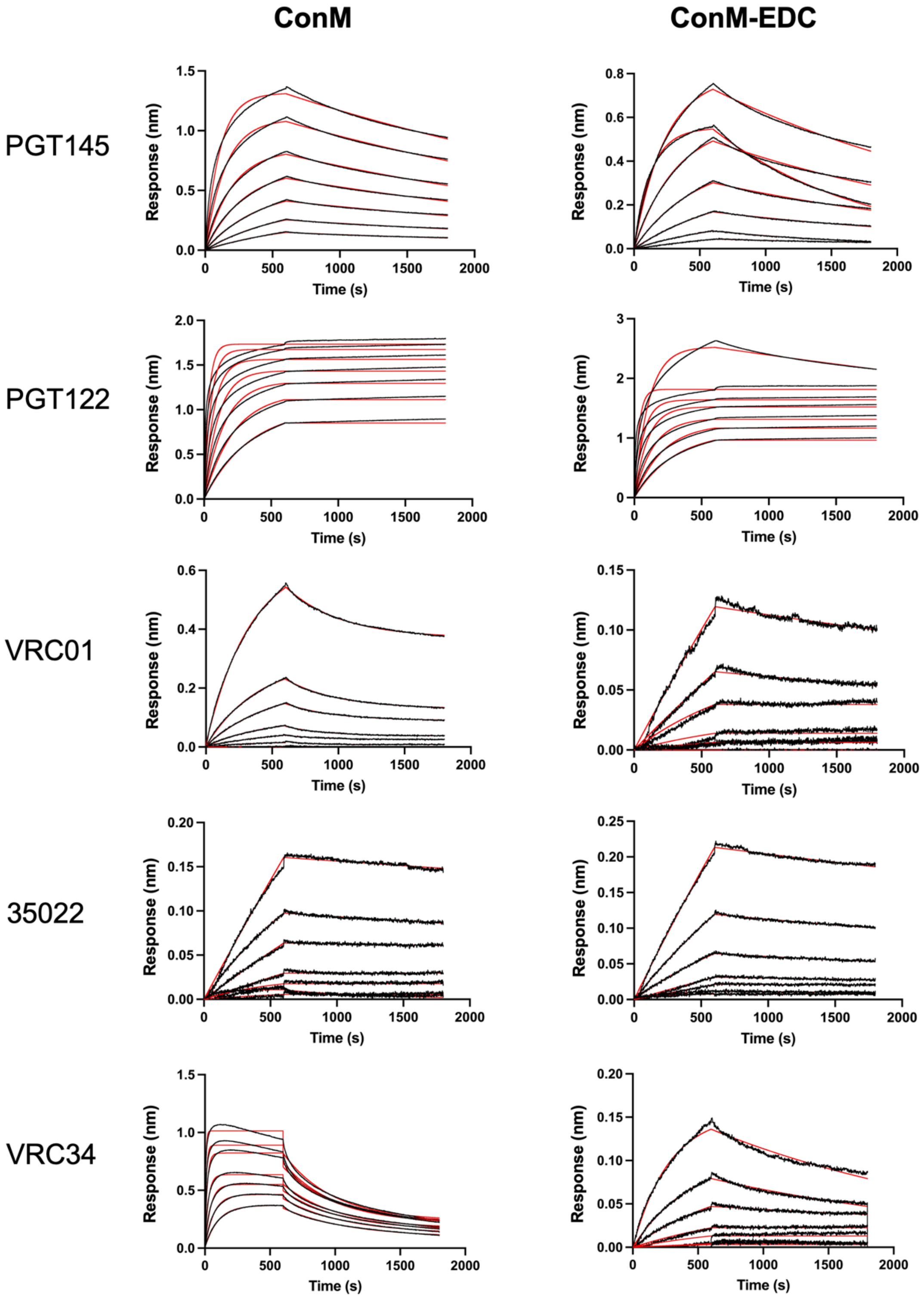

Supplementary Figure 4. Impact of EDC on binding of bNAbs to ConM and ConM-EDC trimers assayed by biolayer interferometry, associated with Table 1. The GMP trimers in this study are untagged, thus bnAb Fab fragments were immobilized to anti-human Fab-CHI biosensors, and binding was analyzed at a range of SOSIP concentrations (2000, 1000, 500, 250, 125, 62.5, 31.25 nM). The aligned and reference-subtracted binding response (colored traces) was fitted to a 1:1 binding model (red lines) to determine kinetic parameters. SOSIP concentrations which gave poor fits (R^2<0.95) were not used for the kinetic analysis. In each case the final values were the average of at least three trimer concentrations.

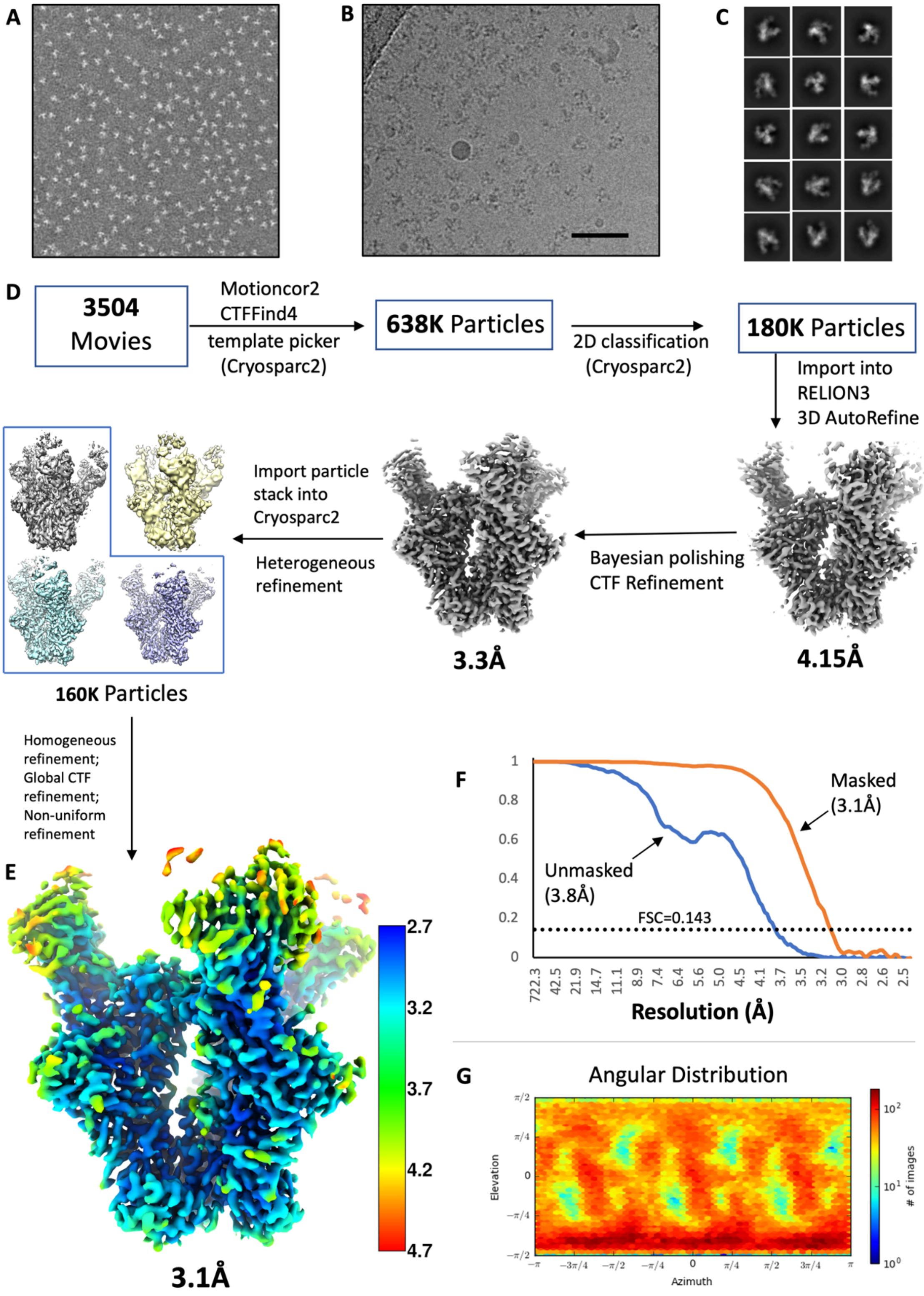

Supplementary Figure 5. Cryo-EM data processing workflow and reconstruction for unmodified ConS, associated with Figure 2. **A)** Representative micrograph from negative stain EM of unliganded (apo) ConS, collected at 120 keV on a Tecnai Spirit. **B)** Motion-corrected cryo-EM micrograph from Titan Krios (1.03 Å/pixel). **C)** Select 2D classes from Cryosparc v2. **D)** Data-processing workflow. **E)** Final reconstruction and local resolution, calculated in Cryosparc v2. Color scale represents local resolution in Angstrom (Å). **F)** Fourier shell correlation. Reported resolutions are according the 0.143 FSC gold standard. **G)** Angular distribution plot of final reconstruction.

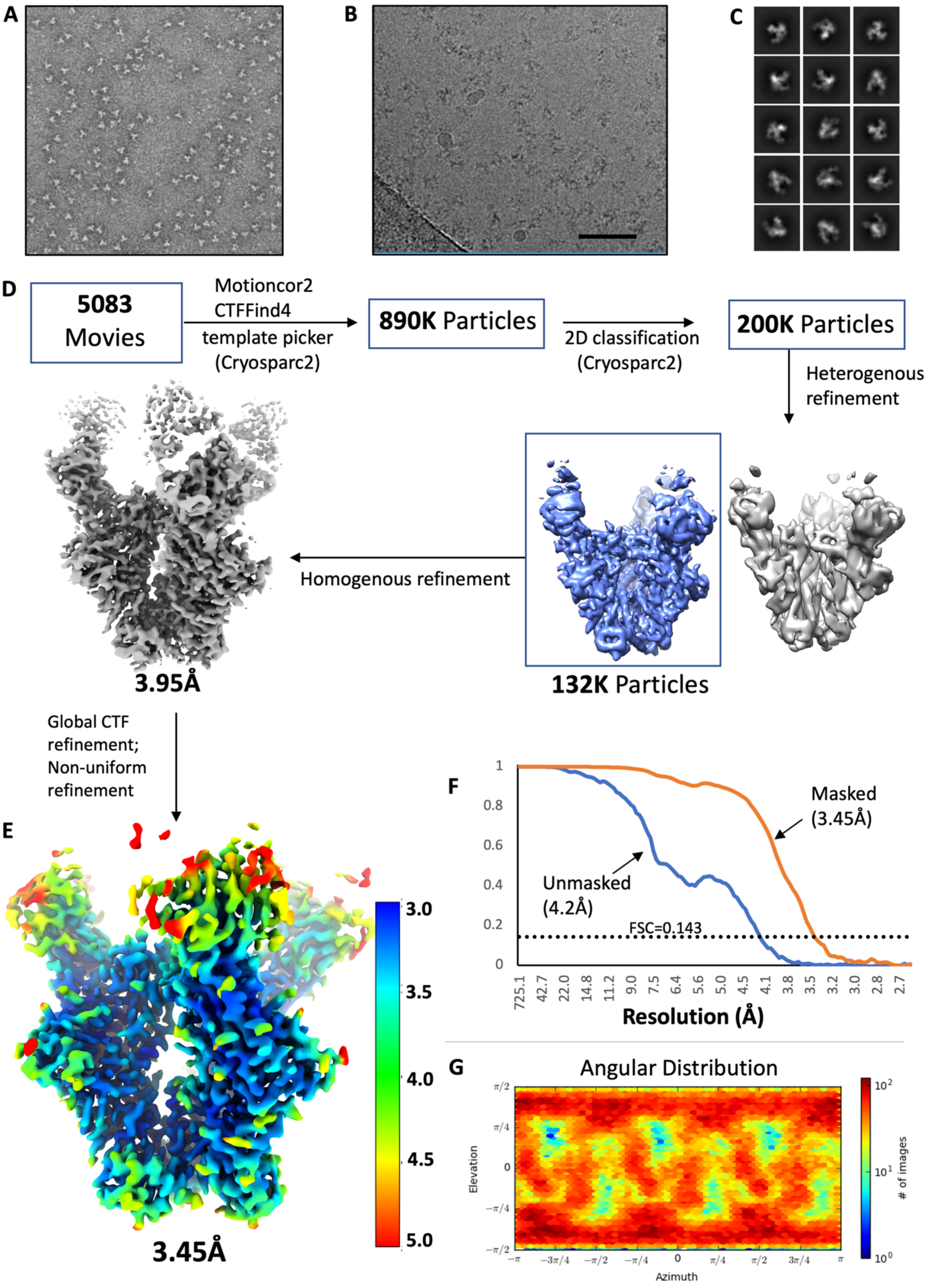

Supplementary Figure 6. Cryo-EM data processing workflow and reconstruction for EDC-crosslinked ConS associated with Figure 2. Same as in Figure S5.

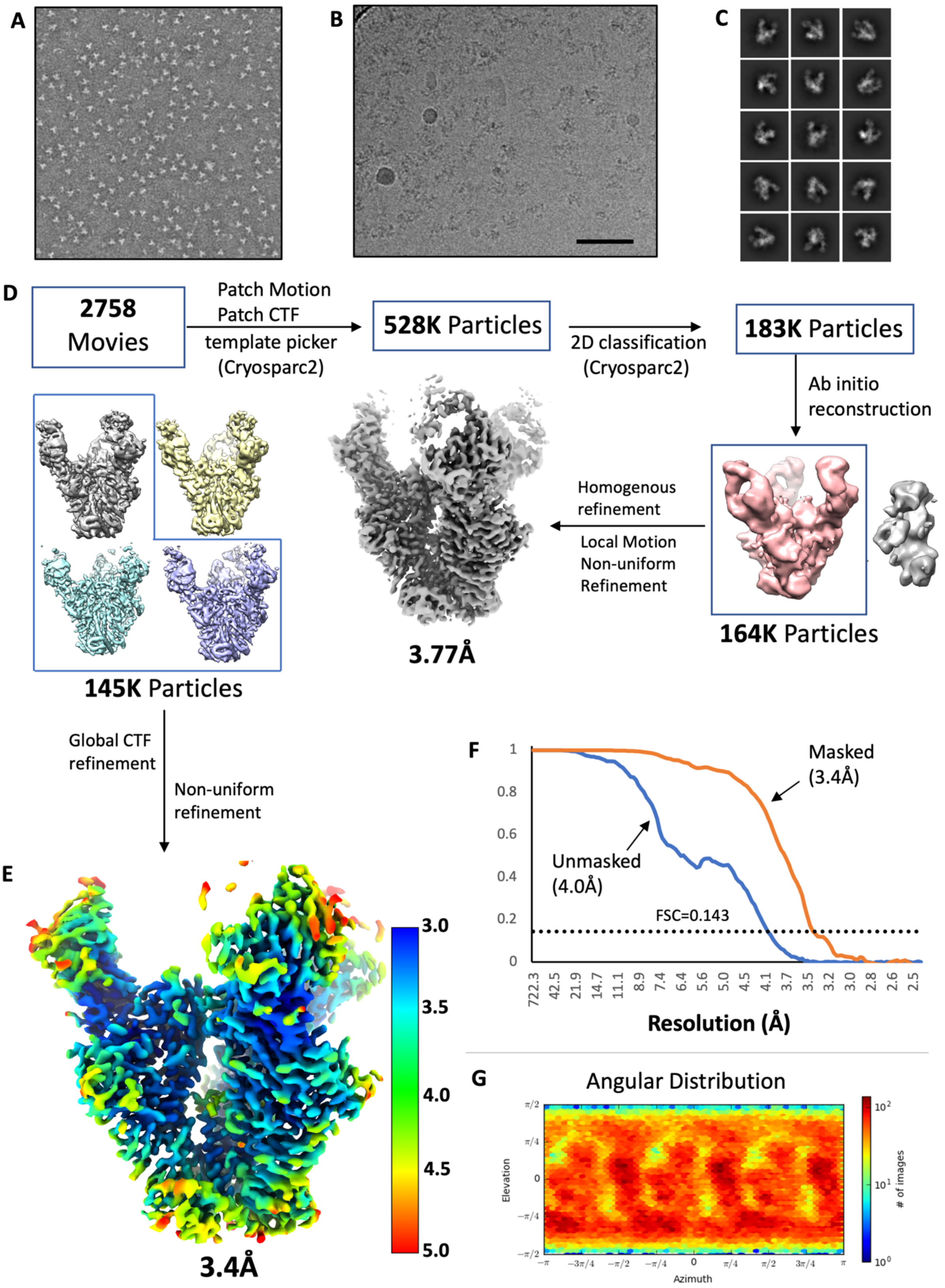

Supplementary Figure 7. Cryo-EM data processing workflow and reconstruction for ConM associated with Figure 2. Same as in Figure S5.

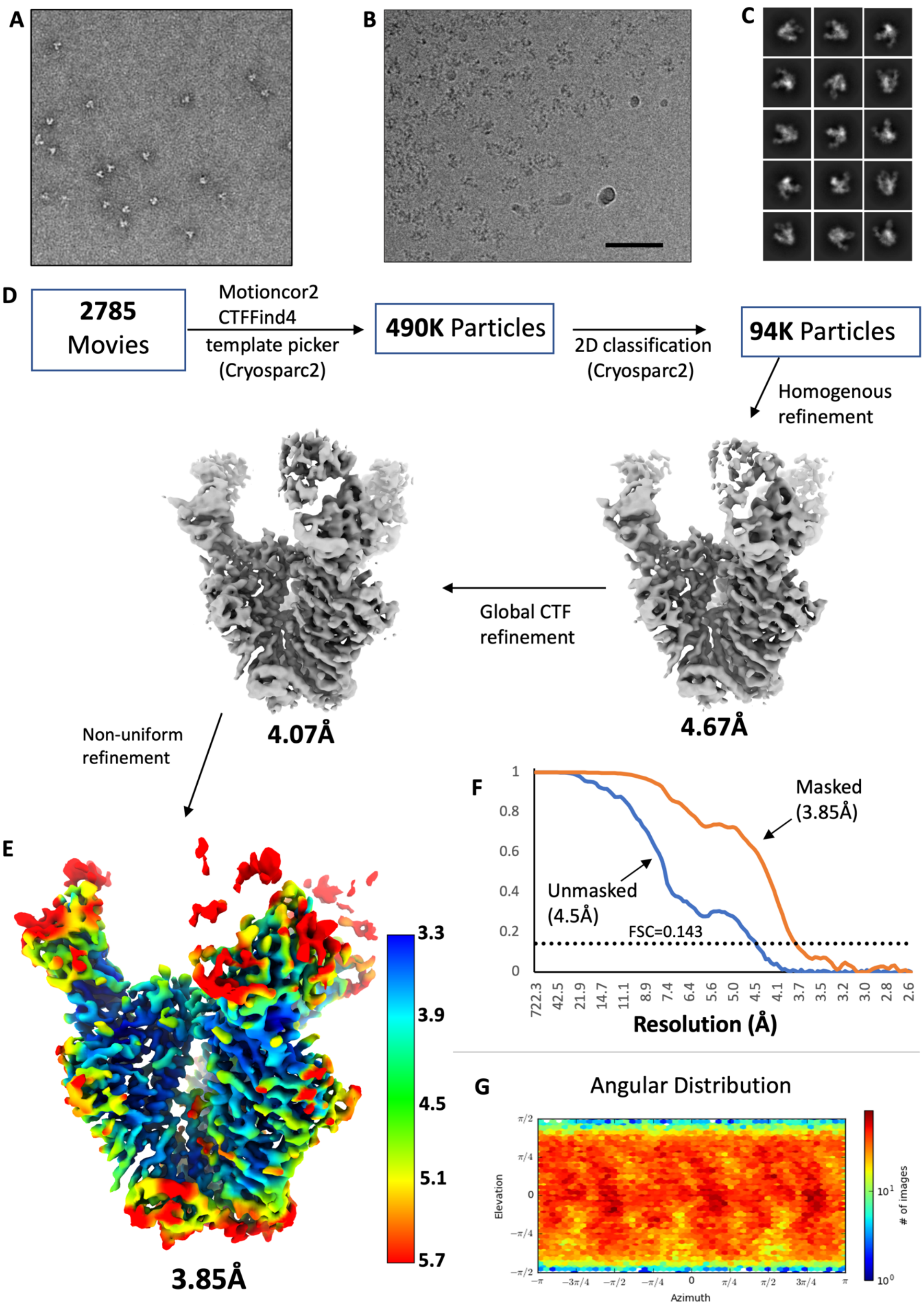

Supplementary Figure 8. Cryo-EM data processing workflow and reconstruction for EDC cross-linked ConM associated with Figure 2. Same as in Figure S5.

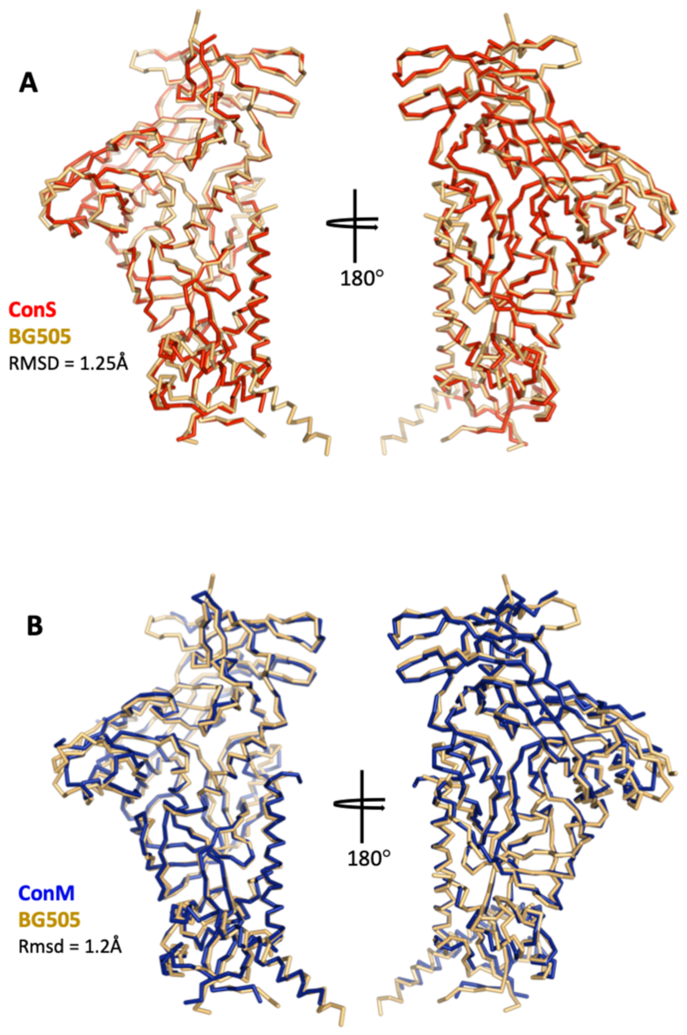

Supplementary Figure 9. **Structural comparisons of ConM and ConS with BG505.**
A) Superimposition of a single protomer of the ConS-PGT122 cryo-EM structure with the BG505-PGT122-35022 X-ray structure (PDB 4TVP). Shown is a ribbon representation of the Cα chain trace. The Cα RMSD is 1.25Å.
B) Same as in (**A**) but comparing BG505 with the ConM-PGT122 cryo-EM structure. The Cα RMSD is 1.2Å.

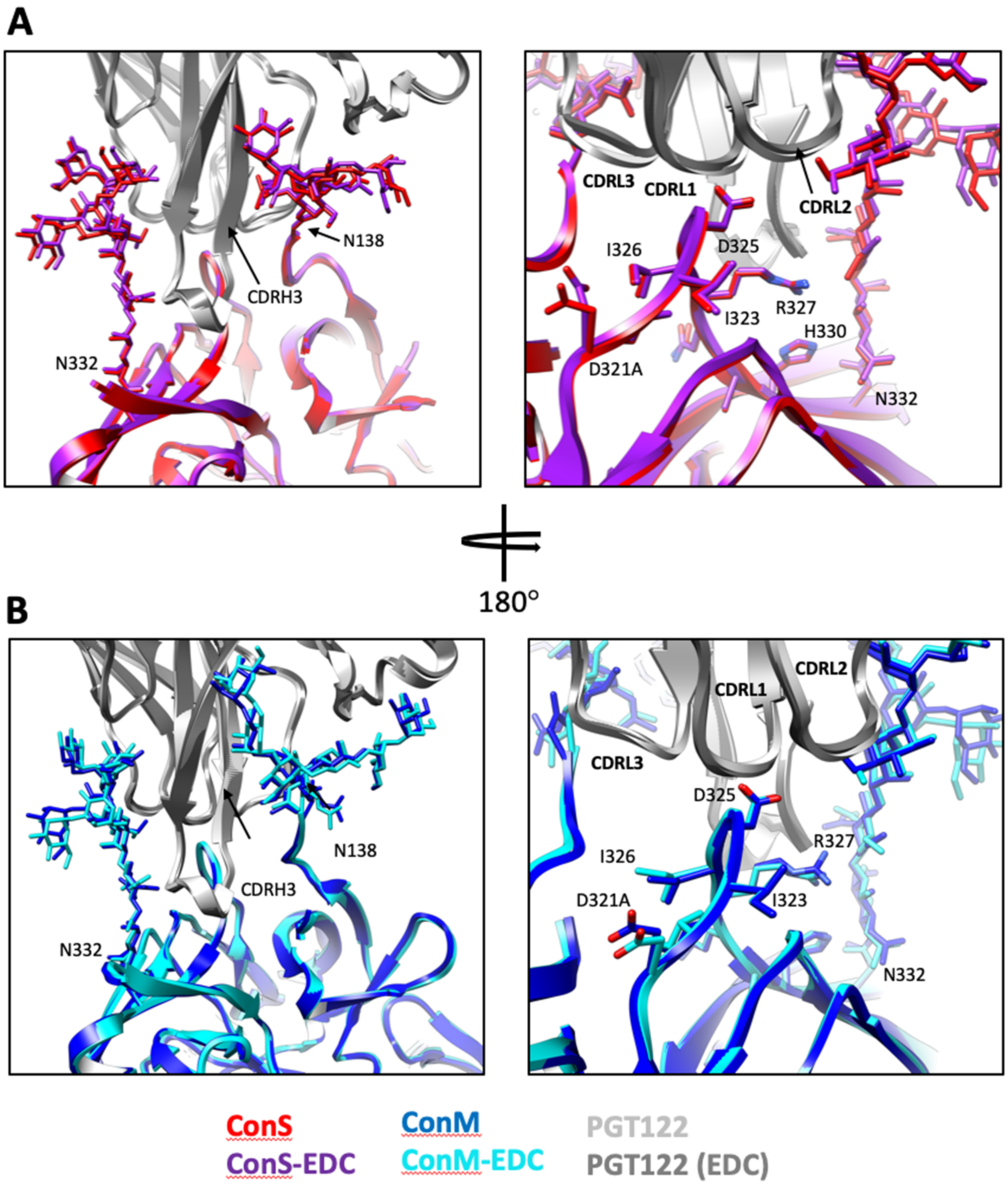

Supplementary Figure 10. **Impact of cross-linking on the PGT122 epitope**. **A)** Superimposition of ConS (red) and ConS-EDC (purple), showing zoomed-in view of PGT122 (grey) binding site. Glycans at N332 and N138 are also shown, which were highly-ordered in the cryo-EM structure. At right the view is rotated 180° and side chains of residues directly in contact with antibody are shown as sticks, including the $G_{324}DIR_{327}$ motif. **B)** Same as in **A**), but for ConM and ConM-EDC.

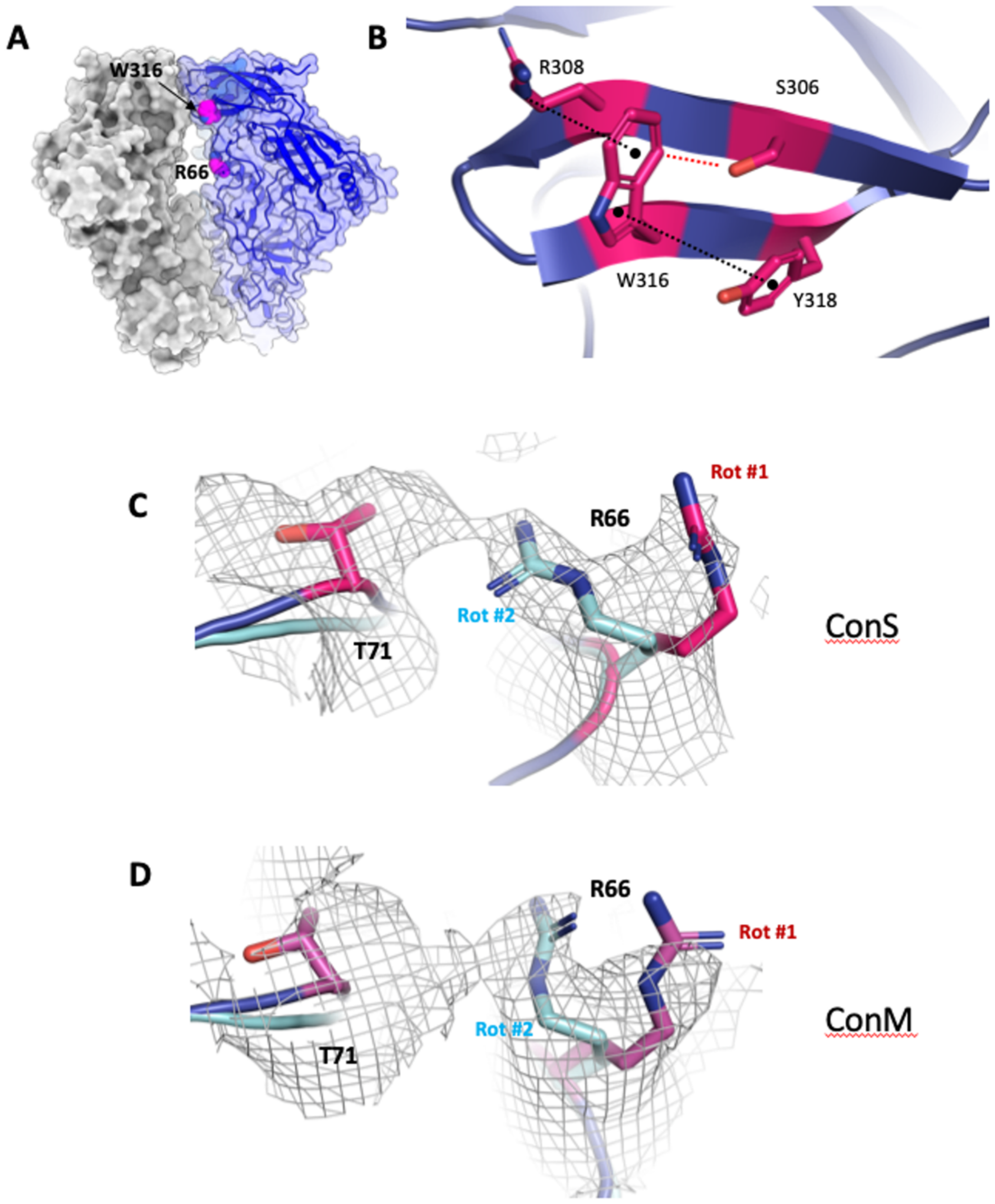

Supplementary Figure 11. Structural details of stabilizing mutations A316W and H66R present in ConS and ConM. **A)** Surface representation of ConS structure, with ribbon representation of one protomer shown. The W316 and R66 side chains are shown as magenta spheres. **B)** Zoomed in view of W316 and the base of the V3 loop in the ConS structure, highlighting a hydrophobic stacking interaction with Y318, a cation-pi interaction with R308, and a possible weak H-bond with S306. **C)** Structure and cryo-EM density of two possible rotamers for the R66 side chain in ConS. R66 is modelled as rotamer #1 in both deposited ConS and ConM cryo-EM structures, but rotamer #2 is equally likely. In this conformation, R66 would probably form stabilizing contacts with T71. **D)** Same as in **C)**, but for ConM cryo-EM structure.

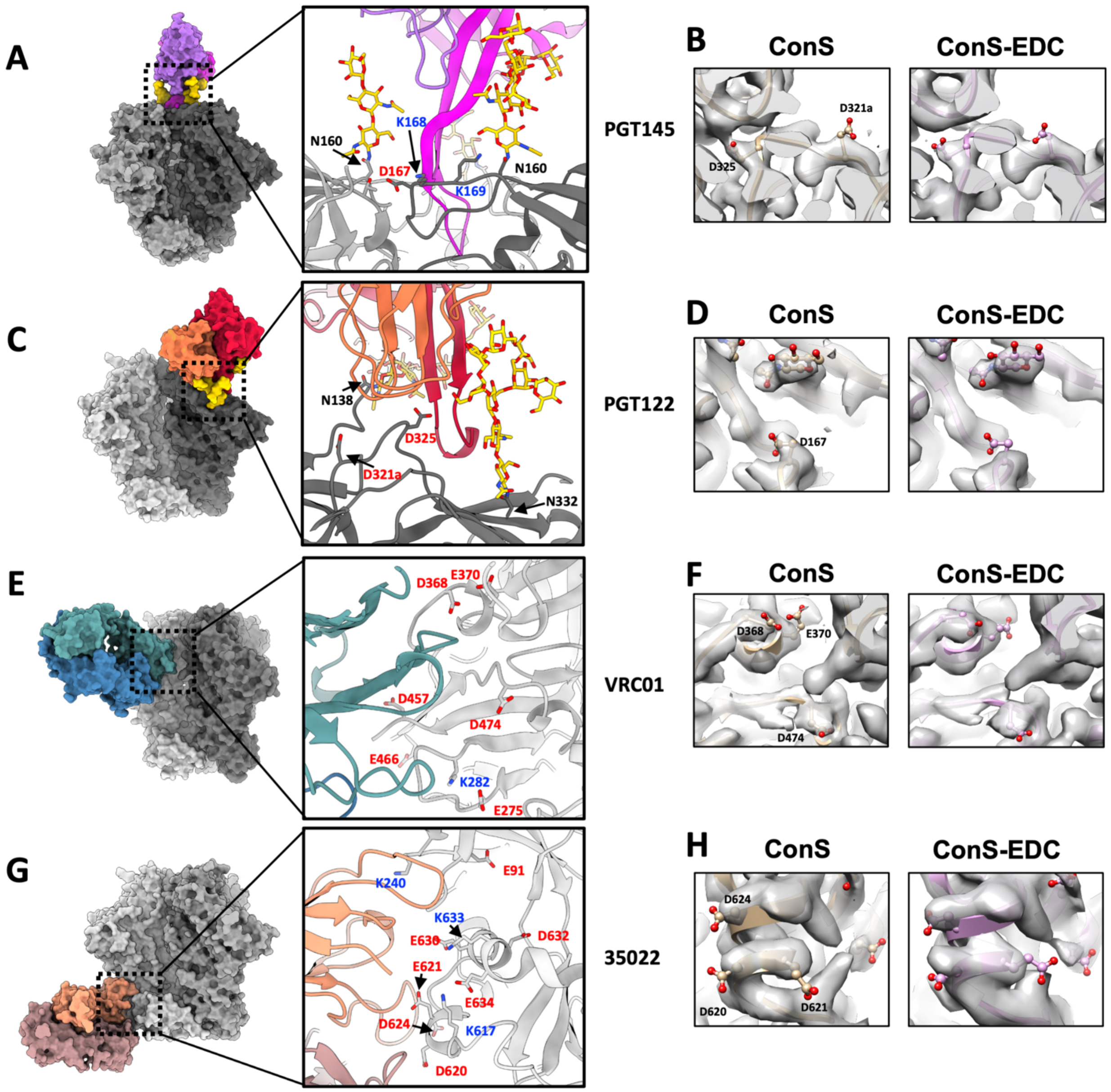

Supplementary Figure 12. Structures of select bnAb epitopes and exposure of glutamate, aspartate, and lysine side chains. **A)** BG505-PGT145 cryo-EM structure (PDB 5V8L). PGT145 binding is to a large extent driven by glycan contacts. **B)** Zoomed in view of select Glu and Asp residues within PGT145 epitope, highlighting the absence of extra cryo-EM density at these positions in the ConS-EDC reconstruction, which could result from glycine quenching of the crosslinking reaction. However, although not directly observed in the cryo-EM structures, a fraction of exposed Glu and Asp side chains could have been modified by EDC crosslinking that would impact antibody binding. **C)** ConS-PGT122 cryo-EM structure (this paper), showing, as with PGT145, a glycan-dependent epitope minimally modified by EDC. **D)** Same as in B, for the PGT122 epitope. **E)** ConS cryo-EM structure with VRC01 docked in, based on PDB 6V8X. **F)** Same as B, for the VRC01 epitope. **G)** ConS cryo-EM structure with docked 35022, based on PDB 5W6D. **H)** Same as in B, for the 35022 epitope.

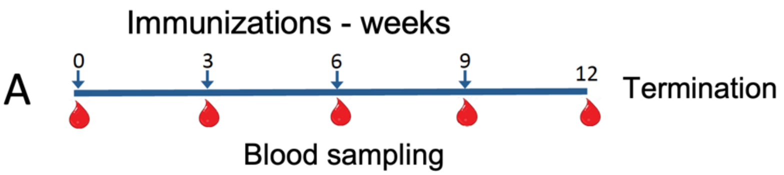

B

Group number	Test item	Concentration (mg/mL)	Dose and volume (µL)	Dose and volume/site (mL)	No. of rabbits
1	ConM + MPLA	0.2 ConM + 1 MPLA	500 ConM + 500 MPLA	0.5	6
2	ConM-EDC + MPLA	0.1 ConM-EDC + 1 MPLA	1000 ConM-EDC + 500 MPLA	0.75	6
3	ConS + MPLA	0.2 ConS + 1 MPLA	500 ConS + 500 MPLA	0.5	6
4	ConS-EDC + MPLA	0.1 ConS-EDC + 1 MPLA	1000 ConS-EDC + 500 MPLA	0.75	6
5	ConM + vehicle	0.2 ConM + 0.9% NaCl	500 ConM + 500 0.9% NaCl	0.5	6

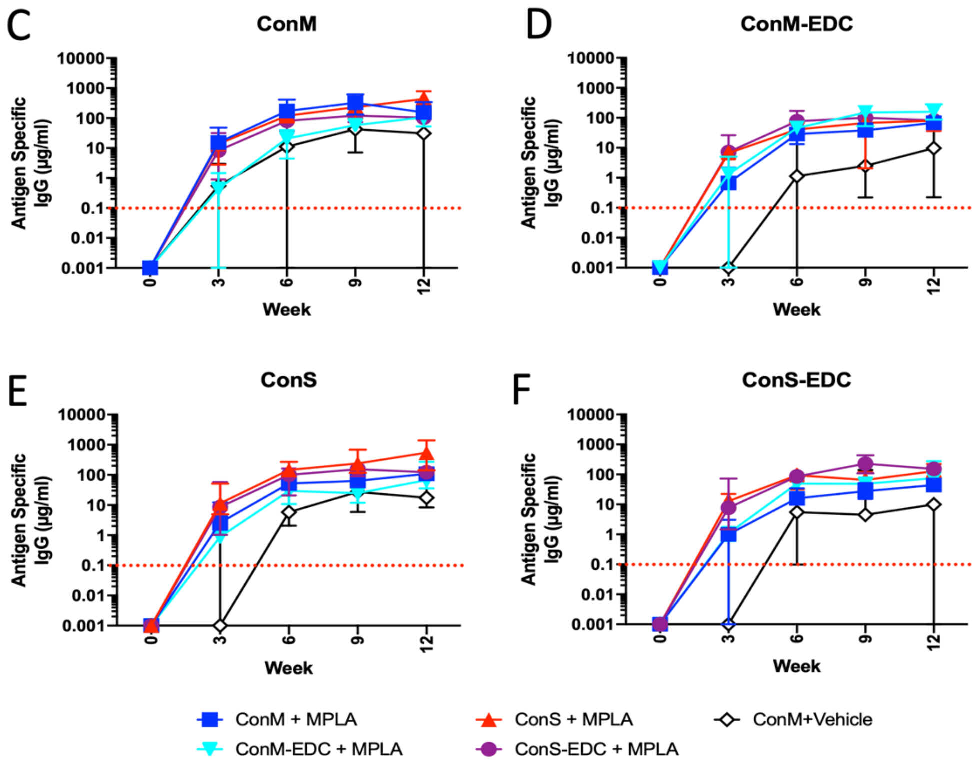

Supplementary Figure 13. **Rabbit IgG responses to antigen in MPLA immunization for toxicity study**. **A**) immunization and sampling regimen where arrows represent immunizations and red drops represent bleeds. The protocol was terminated at week 12. **B**) Schedule of dosing and volumes, animals were injected at 2 sites per administration. Volumes differ between unmodified and EDC cross-linked administrations due to differing starting concentrations of GMP product. **C-F**) IgG purified from sera from rabbits immunized as in **A**) were titrated onto plates coated with: **C**) ConM, **D**) ConM-EDC, **E**) ConS, **F**) ConS-EDC, and results reported as binding curves in µg/mL IgG.

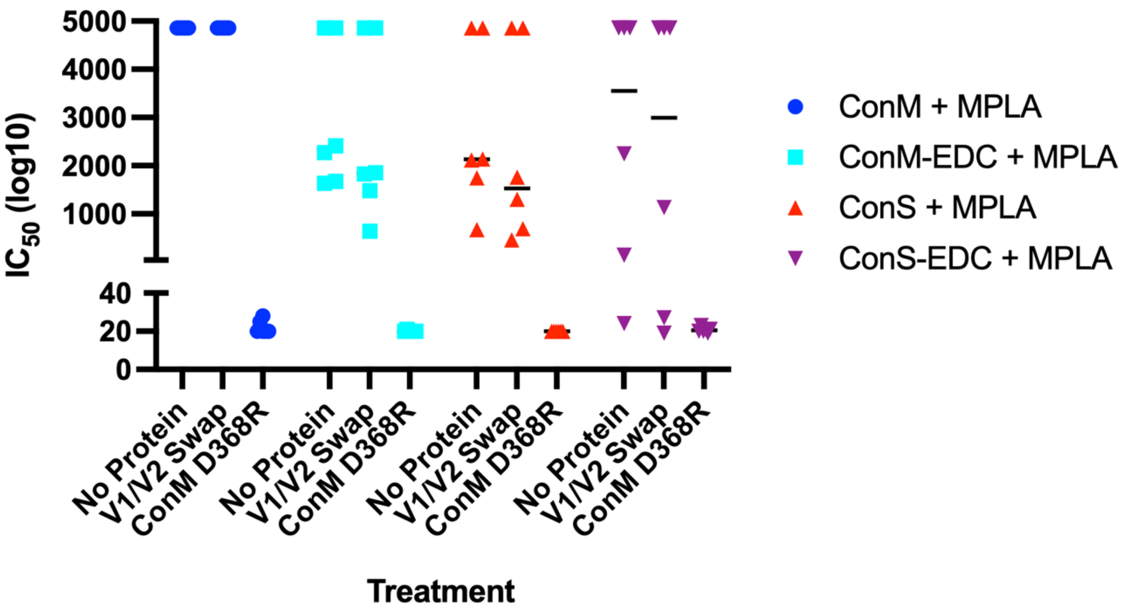

Supplementary Figure 14. **Neutralization depletion by soluble trimer implicates V1V2 loop specificity.** Week 12 sera from trimer-immunized rabbits (Supplementary Figure 12) were preincubated with soluble ConM SOSIP.v7 trimer with or without V1V2 loops swapped for the BG505 sequence and containing the D368R mutation to prevent CD4 binding on the target TZM-bl cells, added to ConM IMC and incubated with HeLa TZM-bl cells, and IC_{50} values calculated.

Supplemental Table 1 – Cryo-EM data collection parameters & model statistics

	**ConM_PGT122**<br>PDB: 7LXM<br>EMDB: EMD-23571	**ConM-EDC_PGT122**<br>PDB: 7LXN<br>EMDB: EMD-23572	**ConS_PGT122**<br>PDB: 7LX2<br>EMDB: EMD-23564	**ConS-EDC_PGT122**<br>PDB: 7LX3<br>EMDB: EMD-23565
**Data Collection**				
Microscope	Titan Krios	Titan Krios	Titan Krios	Titan Krios
Detector	Gatan K2 Summit	Gatan K2 Summit	Gatan K2 Summit	Gatan K2 Summit
Voltage (kV)	300	300	300	300
Pixel size (Å)	1.026	1.026	1.026	1.026
Defocus range (μm)	-1.0 to -2.2	-1.0 to -2.2	-1.0 to -2.2	-1.0 to -2.2
Total electron dose ($e^-/Å^2$)	50	50	50	50
Dose rate ($e^-/Å^2$/sec)	5.2	5.2	4.76	4.72
Frames per exposure	48	48	42	53
**Data Processing**				
Total micrograph movies	2758	2785	3504	5083
Particle images in map	144,741	91,987	161,340	132,156
Symmetry imposed	C3	C3	C3	C3
Map resolution (FSC=0.143; Å)	3.41	3.85	3.12	3.45
Map sharpening B-factor ($Å^2$)	-90.5	-141.6	-105.2	-134.1
**Model Refinement**				
No. of atoms in deposited model				
gp120	10,350	10,350	10,083	10,083
gp41	2850	2850	2598	2598
PGT122	5592	5592	5592	5592
Glycans	2028	2028	1881	1881
RMS deviations				
Bond lengths (Å)	0.019	0.02	0.02	0.02
Bond angles (°)	1.717	1.707	1.633	1.65
Validation				
MolProbity Score	0.89	0.94	0.88	0.84
Clashscore	1.49	1.81	1.43	1.21
EMRinger Score	2.38	2.59	2.98	2.78
Poor rotamers (%)	0.52	1.19	0.15	0.1
Ramachandran Plot				
Favored (%)	98.54	98.58	98.8	99.12
Allowed (%)	1.2	1.29	1.19	0.88
Outliers (%)	0.26	0.13	0	0
Average B-factor	59.1	91.6	35.9	59

Potential Crosslinks

ConS Intra-protomer		ConS Inter-protomer		ConM Intra-protomer		ConM Inter-protomer	
D47	K46	D167.A	K169.C	D47	K46	D167.B	K169.E
D47	K487			D47	K487	E654.B	K601.D
D83	K227			E64	K207	E659.B.	K601.D
E91	K487			E83	K227		
D107	K574			E91	K487		
D113	K117			D107	K574		
D167	K168			D113	K117		
D180	K421			D133	K155		
D230	K232			E153	K178		
D230	K240			D167	K168		
E267	K231			D180	K421		
E268	K231			D230	K232		
E268	K232			D230	K240		
E269	K232			E267	K231		
E269	K348			E268	K231		
E275	K282			E268	K232		
E275	K97			E269	K232		
E351	K347			E269	K348		
E351	K348			E275	K282		
E370	K421			E275	K97		
E466	K356			E290	K340		
E492	K46			E351	K347		
E492	K490			E351	K348		
E621	K617			E370	K421		
E630	K633			E466	K356		
D632	K46			E492	K46		
E634	K617			E492	K490		
				E584	K588		
				E621	K617		
				E630	K633		
				D632	K46		
				E634	K617		

Observed Crosslinks (all intra-protomer)

ConS		ConM	
E268	K232	E267	K231
E269	K348	E268	K232
E293	K337	E290	K340
E351	K348	E485	K231
E632	K46		
E634	K617		

Supplementary Table 2. Summary of potential and observed crosslinks in ConS and ConM. A separation distance of ≤6Å between Glu/Asp and Lys side chains was used to define potential crosslinks, in addition to geometric constraints as observed by the structure. See Methods for determination of observed crosslinks.
