# Supplementary material for: Profound structural conservation of chemically cross-linked HIV-1 envelope glycoprotein experimental vaccine antigens
Source: NPJ Vaccines. 2023 Jul 13;8:101. doi: 10.1038/s41541-023-00696-w (PMC10345191; doi:10.1038/s41541-023-00696-w)
Supplement: Supplementary file 2 — REPORTING SUMMARY [file 41541_2023_696_MOESM2_ESM.pdf]

nature portfolio

Corresponding author(s): Greg Martin: gmartin@scripps.edu, Quentin Sattentau: quentin.sattentau@path.ox.ac.uk Andrew Ward: andrew@scripps.edu

Last updated by author(s): 19th April 2023

Reporting Summary

Nature Portfolio wishes to improve the reproducibility of the work that we publish. This form provides structure for consistency and transparency in reporting. For further information on Nature Portfolio policies, see our Editorial Policies and the Editorial Policy Checklist.

Statistics

For all statistical analyses, confirm that the following items are present in the figure legend, table legend, main text, or Methods section.

n/a	Confirmed	
☐	☒	The exact sample size (*n*) for each experimental group/condition, given as a discrete number and unit of measurement
☐	☒	A statement on whether measurements were taken from distinct samples or whether the same sample was measured repeatedly
☐	☒	The statistical test(s) used AND whether they are one- or two-sided<br>*Only common tests should be described solely by name; describe more complex techniques in the Methods section.*
☒	☐	A description of all covariates tested
☐	☒	A description of any assumptions or corrections, such as tests of normality and adjustment for multiple comparisons
☐	☒	A full description of the statistical parameters including central tendency (e.g. means) or other basic estimates (e.g. regression coefficient) AND variation (e.g. standard deviation) or associated estimates of uncertainty (e.g. confidence intervals)
☐	☒	For null hypothesis testing, the test statistic (e.g. *F*, *t*, *r*) with confidence intervals, effect sizes, degrees of freedom and *P* value noted<br>*Give P values as exact values whenever suitable.*
☒	☐	For Bayesian analysis, information on the choice of priors and Markov chain Monte Carlo settings
☒	☐	For hierarchical and complex designs, identification of the appropriate level for tests and full reporting of outcomes
☒	☐	Estimates of effect sizes (e.g. Cohen's *d*, Pearson's *r*), indicating how they were calculated

Our web collection on statistics for biologists contains articles on many of the points above.

Software and code

Policy information about availability of computer code

Data collection	Leginon software (version 3.5) was used for automated collection of EM data. For collection of biolayer interferometry data, Octet Data Acquisition 9.0.0.8 (ForteBio) software was used.
Data analysis	Micrograph movie frames were aligned and dose-weighted using MotionCor2. CTF estimation was performed with CTFFind4. Single particle data processing was performed with both cryoSPARC v3.3 and and RELION-3.0. For cryo-EM structure modelling, UCSF Chimera v1.16, Coot v0.9.8.6, RosettaCM, Rosetta Relax, PyMol v1.20, and PHENIX v1.20.1 were used. For biolayer interferometry data analysis, ForteBio Data Analysis v9.0 (ForteBio) was used. Graph preparation and statistical analysis were carried out with Graphpad Prism 7.

For manuscripts utilizing custom algorithms or software that are central to the research but not yet described in published literature, software must be made available to editors and reviewers. We strongly encourage code deposition in a community repository (e.g. GitHub). See the Nature Portfolio guidelines for submitting code & software for further information.

Data

Policy information about availability of data

All manuscripts must include a data availability statement. This statement should provide the following information, where applicable:

- Accession codes, unique identifiers, or web links for publicly available datasets
- A description of any restrictions on data availability
- For clinical datasets or third party data, please ensure that the statement adheres to our policy

The final cryo-EM reconstructions and the resulting structural models have been deposited into the Protein Data Bank (PDB) the Electron Microscopy Data Bank (EMDB) under the following accession codes: ConS: PDB 7LX2, EMDB 23564; ConS-EDC: PDB 7LX3, EMDB 23565; ConM: PDB 7LXM, EMDB 23571; ConM-EDC: PDB 7LXN, EMDB 23572. Other data supporting this study are available from the corresponding authors upon reasonable request.

Research involving human participants, their data, or biological material

Policy information about studies with human participants or human data. See also policy information about sex, gender (identity/presentation), and sexual orientation and race, ethnicity and racism.

Reporting on sex and gender	n/a
Reporting on race, ethnicity, or other socially relevant groupings	n/a
Population characteristics	n/a
Recruitment	n/a
Ethics oversight	n/a

Note that full information on the approval of the study protocol must also be provided in the manuscript.

Field-specific reporting

Please select the one below that is the best fit for your research. If you are not sure, read the appropriate sections before making your selection.

☒ Life sciences ☐ Behavioural & social sciences ☐ Ecological, evolutionary & environmental sciences

For a reference copy of the document with all sections, see nature.com/documents/nr-reporting-summary-flat.pdf

Life sciences study design

All studies must disclose on these points even when the disclosure is negative.

Sample size	Group sizes for figures 1 and 4 were determined by pilot studies to estimate effect size difference between groups which was then used to determine experimental group size in the final experiments.
Data exclusions	No data were excluded from the analysis.
Replication	All replications resulted in reproducible results.
Randomization	For in vivo experiments, all groups of mice were matched by age and sex.
Blinding	n/a

Reporting for specific materials, systems and methods

We require information from authors about some types of materials, experimental systems and methods used in many studies. Here, indicate whether each material, system or method listed is relevant to your study. If you are not sure if a list item applies to your research, read the appropriate section before selecting a response.

Materials & experimental systems

n/a	Involved in the study
☐	☒ Antibodies
☐	☒ Eukaryotic cell lines
☒	☐ Palaeontology and archaeology
☐	☒ Animals and other organisms
☒	☐ Clinical data
☒	☐ Dual use research of concern
☒	☐ Plants

Methods

n/a	Involved in the study
☒	☐ ChIP-seq
☒	☐ Flow cytometry
☒	☐ MRI-based neuroimaging

Antibodies

Antibodies used

VRC0144, PGT14545, 2G1246, PGT12245, 19b47, 3502248, ACS20249 and VRC34 15e47, F10547, 17b31, 19b and PG1652 were all produced, verified and provided by the source laboratories (see acknowledgments section).
Secondary antibodies for use in ELISA and western blot were obtained from and verified by commercial sources (see methods).

Validation

n/a

Eukaryotic cell lines

Policy information about cell lines and Sex and Gender in Research

Cell line source(s)

HEK293T cells (ATCC Cat. No. CRL-3216)
HEK293F cells (ThermoFisher Cat. No. R79009)
TZM-bl HeLa cells obtained from National Institute for Biological Standards and Controls (Cat No. ARP5011)
CHO K1 cells were derived in-house from ECACC: CHO-K1 (ECACC 85051005) (ATCC: CCL 61)

Authentication

None of the cell lines used were authentificated

Mycoplasma contamination

All cell lines tested negative in-house for mycoplasma.

Commonly misidentified lines
(See ICLAC register)

n/a

Animals and other research organisms

Policy information about studies involving animals; ARRIVE guidelines recommended for reporting animal research, and Sex and Gender in Research

Laboratory animals

Female inbred mice of the BALB/c strain 6 - 8 weeks old were bred in-house (Oxford University) under UK home office guidelines. Rabbit studies using 11 week old New Zealand White specific pathogen-free animals of equal numbers of male and female sex were carried out at Covance Inc.

Wild animals

No wild animals were used in this study

Reporting on sex

Equal numbers of male and female rabbits were used in this study. Only female mice were used in this study.

Field-collected samples

No field collected samples were used in this study

Ethics oversight

Animal research using rabbits and mice was carried out in full accordance with local and national ethical guidelines. All protocols for breeding and procedures with mice were approved by the Home Office UK, under the Animals (Scientific Procedures) Act 1986 and UK Home Office Project License PPL3003421. Rabbit studies were carried out at Covance Inc.

Note that full information on the approval of the study protocol must also be provided in the manuscript.
